# Astaxanthin supplementation in Arabian racing horses mitigates oxidative stress and inflammation in peripheral blood mononuclear cells through enhanced mitophagy

**DOI:** 10.1038/s41598-025-93661-7

**Published:** 2025-04-26

**Authors:** Beata Giercuszkiewicz-Hecold, David Pajuelo, Zofia Steczkiewicz, Anna Cywinska, Krzysztof Marycz

**Affiliations:** 1 Doctoral School, Warsaw University of Life Sciences-SGGW, Nowoursynowska 159c, 02-776 Warsaw, Poland; 2 Faculty of Health Sciences, Universidad Europea de Valencia, 46010 Valencia, Spain; 3 Department of Experimental Biology, Faculty of Biology and Animal Science, Wrocław University of Environmental and Life Sciences, Norwida 27B, 50-375 Wrocław, Poland; 4 Department of Basic and Preclinical Sciences, Faculty of Biological and Veterinary Sciences, Nicolaus Copernicus University in Torun, Lwowska 1, 87-100 Toruń, Poland; 5 International Institute of Translational Medicine (MIMT), ul. Jesionowa 11, 55-114 Malin Wisznia Mała, Poland

**Keywords:** Horses, Astaxanthin, Dietary supplementation, Oxidative stress, Inflammation, Mitochondria, PBMC, Cell biology, Molecular biology

## Abstract

Astaxanthin, a strong antioxidant carotenoid, has shown promising features in mitigating inflammation and oxidative stress and so that has been considered as a supplement for high-performance animals. In this study, we aimed to evaluate the effects of astaxanthin on oxidative stress, inflammation, and mitochondrial health in peripheral blood mononuclear cells (PBMC) isolated from Arabian racehorses. Horse-derived peripheral blood mononuclear cells exposed to hydrogen peroxide (H₂O₂) presented increased reactive oxygen species (ROS) accumulation and overexpression of pro-inflammatory cytokines such as IL-1β, IL-6, IFN-γ, and TNF-α. The addition of astaxanthin to cell culture reduced H₂O₂-induced inflammatory response by decreasing the expression levels of all the tested pro-inflammatory cytokines. Moreover, astaxanthin displayed a potential antioxidant response by increasing the expression of genes related to antioxidative defense, such as *NRF1*, *SOD2*, and *GPX*. Interestingly, PBMCs isolated from the horses orally supplemented with astaxanthin increased the expression of the mitophagy-related genes *PINK1* and *PARKIN*. Moreover, genes related to mitochondrial dynamics and energy production, such as *PPARGC1B*,* NDUFA9*, and *MRPL24*, as well as genes associated with mitochondrial function, structure and dynamics, such as *PIGBOS*,* MRLP24*,* PUSL1* and *TFAM* were upregulated in PBMCs isolated from astaxanthin supplemented horses. Altogether, these findings indicate that astaxanthin may be a beneficial dietary supplement for equine health, supporting resilience against oxidative stress and inflammatory challenges, and improving the recovery and performance of racing horses.

## Introduction

Astaxanthin is a naturally occurring carotenoid with potent antioxidant properties, commonly found in marine organisms such as microalgae, salmon, and shrimp. The chemical structure of astaxanthin, which includes conjugated double bonds and hydroxyl groups, allows it to efficiently scavenge free radicals and protect cells from oxidative damage^1,2^. This strong antioxidant capacity has sparked interest in its potential applications in animal nutrition, particularly for high-performance animals such as racehorses^3,4^. Given the high metabolic demand and oxidative stress that racehorses experience during training and competition, incorporating astaxanthin into their diet could offer significant health benefits by mitigating oxidative damage and enhancing recovery. Studies have demonstrated that astaxanthin can reduce markers of oxidative stress and improve overall antioxidant status in animals. For example, a study by Ambati et al. (2014) highlighted the broad-spectrum antioxidant activity of astaxanthin, which includes protecting lipids, proteins, and DNA from oxidative damage^5^. Additionally, it has been shown that dietary astaxanthin had beneficial effects on the antioxidant status of not only race horses, but also for other animals^6–8^.

To investigate the protective effects of astaxanthin against oxidative stress, we used a model involving horse-derived peripheral blood mononuclear cells (PBMCs). In this model, oxidative stress was induced by treating the cells with hydrogen peroxide (H₂O₂), which significantly increases the production of reactive oxygen species (ROS). This method effectively simulates the oxidative stress conditions that cells might encounter in vivo, allowing the investigation of the cellular responses and the potential protective effects of antioxidants like astaxanthin.

Oxidative stress and inflammation are closely linked processes that can detrimentally affect the health and performance of racehorses^9^. During intense physical activity, the production of ROS can overwhelm the body’s antioxidant defenses, leading to cellular damage and the activation of inflammatory pathways. Elevated ROS levels cause oxidative modifications to cellular components such as lipids, proteins, and DNA, which can disrupt normal cellular functions and trigger stress responses. These oxidative modifications are recognized by the immune system as damage signals, leading to the activation of inflammatory pathways^10^. Oxidative stress induces the activation of transcription factors such as nuclear factor-kappa B (NF-κB) and activator protein 1 (AP-1), which are pivotal in regulating the expression of pro-inflammatory cytokines. This activation results in the increased production and release of cytokines such as interleukin-1β (IL-1β), interleukin-6 (IL-6), tumor necrosis factor-alpha (TNF-α), and interferon-gamma (IFN-γ). These pro-inflammatory cytokines play a crucial role in initiating and sustaining the inflammatory response. In racehorses, the systemic release of these cytokines due to oxidative stress can trigger the processes, which in a certain extent, may contribute to the impaired muscle function and recovery but also promotes chronic health issues such as joint diseases and metabolic muscle disorders. Inflammation exceeding certain levels is particularly detrimental for racehorses as it affects overall performance and health^11^. Inflammation in muscle tissues can result in pain, stiffness, and reduced muscle function, making it difficult for horses to perform at their peak levels. Moreover, chronic inflammation can lead to fatigue, slower recovery times, and increased susceptibility to injuries and illnesses. Therefore, it is crucial to manage and balance the triggering of inflammation to ensure the optimal health and performance of racehorses. Effective management of oxidative stress and inflammation in racehorses involves strategies that enhance antioxidant defenses and reduce pro-inflammatory signals. This can be achieved through dietary supplementation with antioxidants such as astaxanthin, which has been shown to reduce oxidative damage and modulate inflammatory responses^3,4^. By reducing oxidative stress and pro-inflammatory signaling, astaxanthin can help maintain muscle health, improve recovery times, and enhance overall performance in racehorses.

Mitophagy, the selective degradation of damaged mitochondria by autophagy, plays a vital role in maintaining mitochondrial quality and cellular health^12^. This process involves the identification and removal of dysfunctional mitochondria, which are then targeted for degradation and recycling within lysosomes. Efficient mitophagy ensures the removal of mitochondria that produce excessive ROS, thereby preventing the accumulation of oxidative stress within cells^12^. By maintaining a population of healthy mitochondria, mitophagy helps to sustain cellular energy production and minimize cellular damage.

In the context of reducing inflammation and oxidative stress, mitophagy is crucial^12,13^. Dysfunctional mitochondria are significant sources of ROS, which can damage cellular components and trigger inflammatory pathways. When damaged mitochondria are not effectively removed, they can lead to increased oxidative stress and the activation of pro-inflammatory signaling cascades, such as the NF-κB pathway, resulting in the production of cytokines like TNF-α, IL-1β, and IL-6. These cytokines further exacerbate inflammation, creating a vicious cycle of oxidative stress and inflammatory responses.

The importance of mitophagy in cellular health and its role in disease prevention has been highlighted in various studies. For instance, studies have shown that enhancing mitophagy can alleviate oxidative stress and inflammation in neurodegenerative diseases and metabolic disorders^14^. Moreover, research indicates that promoting mitophagy can protect against myocardial ischemia-reperfusion injury by reducing oxidative damage and inflammation^15^. In high-performance racehorses, where the energy demands and oxidative stress levels are elevated, enhanced mitophagy can support sustained energy production and stamina, which are critical for optimal performance. By maintaining mitochondrial health through effective mitophagy, racehorses can better manage the increased metabolic demands of intense physical activity. This not only improves their performance but also reduces the risk of oxidative stress-related damage and inflammation, thereby promoting overall health and longevity.

Our study aims to explore the therapeutic potential of astaxanthin in promoting mitophagy as a means to reduce oxidative stress and inflammation in racehorses. By in vitro demonstrating that astaxanthin enhances the expression of genes involved in the mitophagy process, such as PINK1 and PARKIN, we provide evidence for its role in improving mitochondrial quality and cellular resilience. This therapeutic approach highlights the importance of targeting mitochondrial health to mitigate the detrimental effects of oxidative stress and inflammation, thereby supporting the performance and well-being of high-performance racehorses. In our study, we discovered that the number of ROS-positive cells was reduced, the expression of pro-inflammatory cytokines was decreased, and mitophagy was modulated in PBMCs derived from astaxanthin supplemented horses. These findings suggest that astaxanthin not only provides antioxidative and anti-inflammatory benefits but also enhances mitochondrial quality control mechanisms, offering a comprehensive approach to improving the health and performance of racehorses.

## Results

### Evaluation of the in vitro protective effect of astaxanthin against oxidative stress in horse-derived PBMCs

**Fig. 1:**
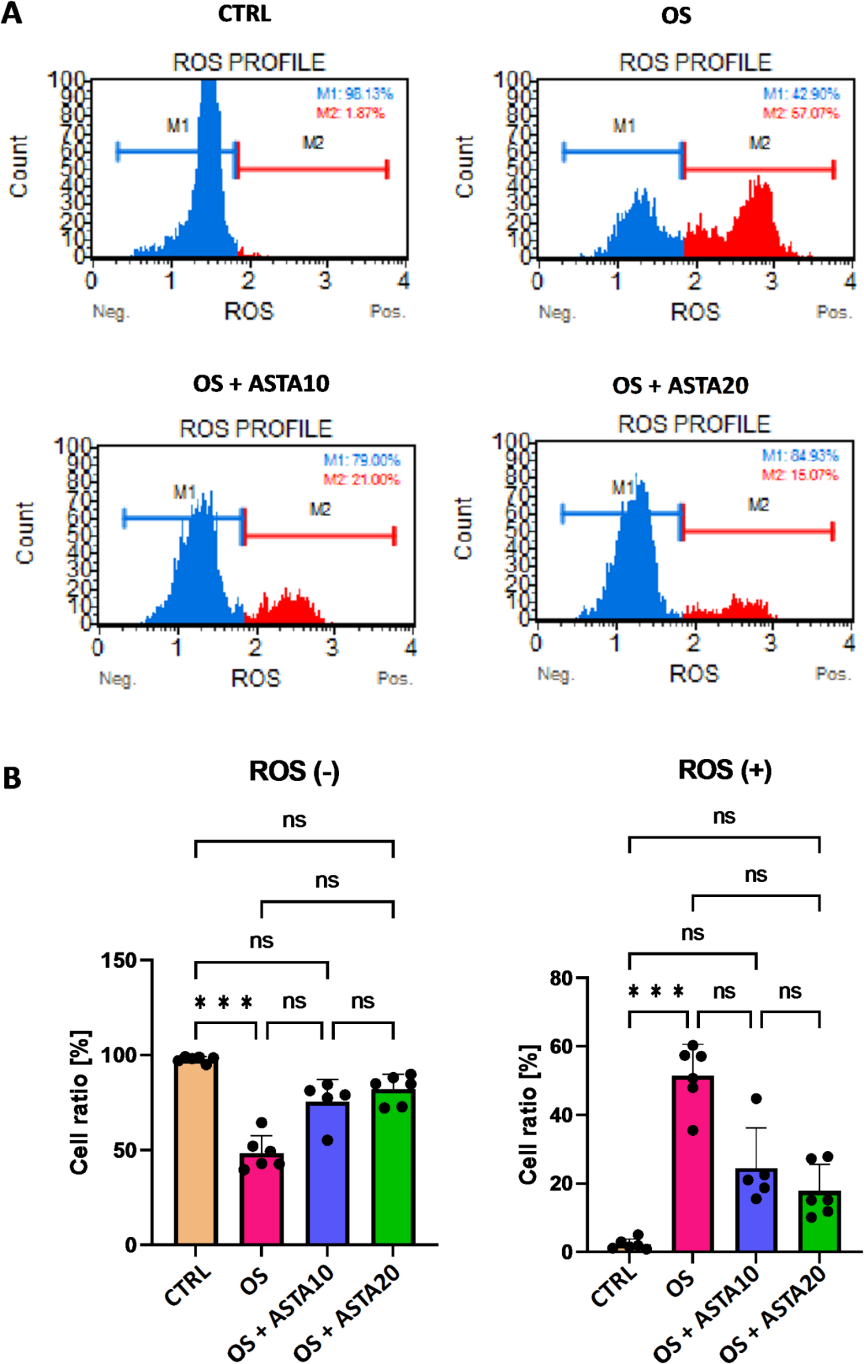
Effect of astaxanthin on the oxidative stress of horse PBMCs. ROS accumulation in control cells (CTRL), oxidative stress-induced cells (OS) and oxidative stress-induced cells treated with astaxanthin at 10 µM (OS + ASTA10) or 20 µM (OS + ASTA20) was tested using the Muse Analyzer Flow Cytometry. (**A**) Flow cytometry histograms show the percentage of ROS-negative (blue, M1) and ROS-positive (red, M2) cells. (**B**) Bar graphs show the statistical analysis of all pairwise comparisons in ROS-negative and ROS-positive cells. Mean and standard deviation are given. Statistical significance is depicted as; *** p-value < 0.001; ns, not significant.

As shown at Fig. 1.A, treatment with H_2_O_2_ (oxidative stress condition, OS) increased the number of ROS-positive cells, while decreased the number of ROS-negative cells. Most importantly, the addition of astaxanthin at either 10 µM (OS + ASTA10) or 20 µM (OS + ASTA20) partially restored the control phenotype by reducing the number of ROS-positive cells and increasing the number of ROS-negative cells (Fig. 1.A). These data were presented in bar graphs and statistically analyzed, showing that H_2_O_2_ significantly increased ROS accumulation in cells (Fig. 1.B). Although astaxanthin decreased the ROS production by reducing the ROS-positive cells and increasing the ROS-negative cells, these changes were not statistically significant (Fig. 1.B).

**Fig. 2:**
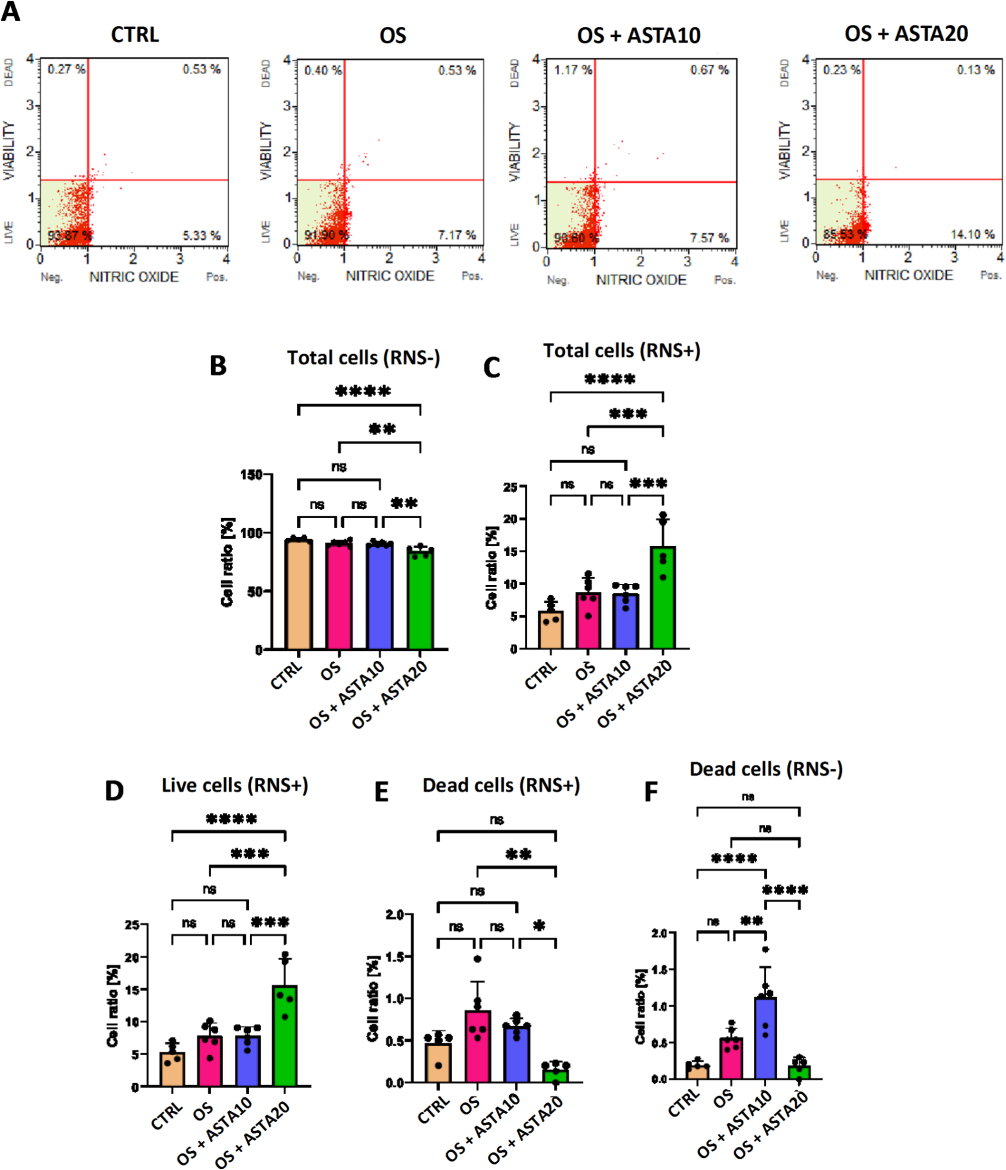
Effect of astaxanthin in the nitrosative stress of horse PBMCs. RNS accumulation in control cells (CTRL), oxidative stress-induced cells (OS) and oxidative stress-induced cells treated with astaxanthin at 10 µM (OS + ASTA10) or 20 µM (OS + ASTA20) was tested using the Muse Analyzer Flow Cytometry. (**A**) Flow cytometry histograms show the percentage of viable and RNS-positive cells. (**B**–**F**) Bar graphs show the statistical analysis of all pairwise comparisons in RNS-negative and RNS-positive cells. Mean and standard deviation are given. Statistical significance is depicted as; * p-value < 0.05, ** p-value < 0.01, *** p-value < 0.001, **** p-value < 0.0001; ns, not significant.

The quantitative analysis of reactive nitrogen species (RNS) revealed that the addition of astaxanthin at 20 µM increased the levels of cellular RNS, as shown by the decrease of RNS-negative cells and the increase of the number of RNS-positive cells (Fig. 2A–C). When the cell population was gated according to the cell viability (live/dead), the accumulation of RNS mentioned above was seen mainly in the live cell population (Fig. 2A,D). When focused on the dead cell population, it has been shown that astaxanthin reduced the number of RNS-positive cells, probably because most of the RNS-positive cells were still alive (Fig. 2A,E,F). Overall, these results suggest that horse-derived PBMCs do not induce RNS production in response to an external oxidative stress induction with H_2_O_2_ and so that, in the tested conditions, astaxanthin increases the nitrosative stress.

**Fig. 3:**
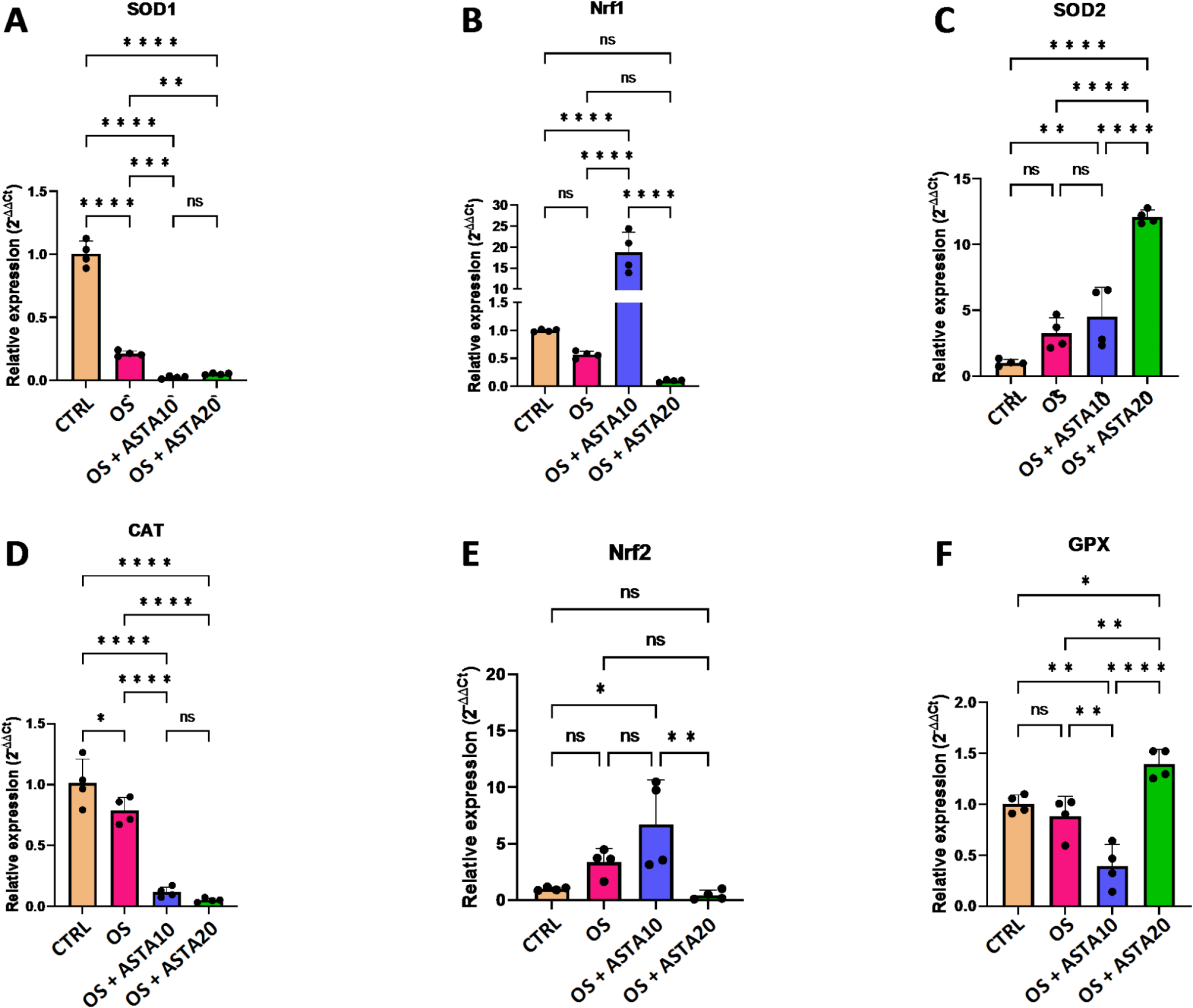
Effect of astaxanthin on the gene expression of oxidative stress markers in horse PBMCs. (**A**–**F**) Analysis of the gene expression levels of selected oxidative stress markers (*SOD1*,* NRF1*,* SOD2*,* CAT*,* NRF2*,* GPX*) in control cells (CTRL), oxidative stress-induced cells (OS) and oxidative stress-induced cells treated with astaxanthin at 10 µM (OS + ASTA10) or 20 µM (OS + ASTA20) by qRT-PCR. Mean and standard deviation are given. Statistical significance is depicted as; * p-value < 0.05, ** p-value < 0.01, *** p-value < 0.001, **** p-value < 0.0001; ns, not significant.

To deeper characterize the antioxidant properties of astaxanthin in horse-derived PBMCs, the cells were challenged with H_2_O_2_ and the expression of genes associated with the detoxification of oxidative stress were evaluated upon the treatment with astaxanthin. As shown in Fig. 3, none of the selected genes (*SOD1*,* NRF1*,* SOD2*,* CAT*,* NRF2*,* GPX*) was induced in the presence of H_2_O_2_. This might result from the sampling time since the gene expression might have been induced earlier and then inhibited once the genes have been transcribed and translated. Most importantly, the treatment with astaxanthin increased the gene expression of *NRF1*, *SOD2*, and *GPX* compared to the H_2_O_2_-treated condition (Fig. 3B,C,F). Altogether, the gene expression analysis indicates that the treatment with astaxanthin increased the expression of genes associated with the detoxification of oxidative stress and might prime the PBMCs to be protected against reactive oxygen species.

### Anti-inflammatory properties of astaxanthin in horse-derived PBMCs

**Fig. 4:**
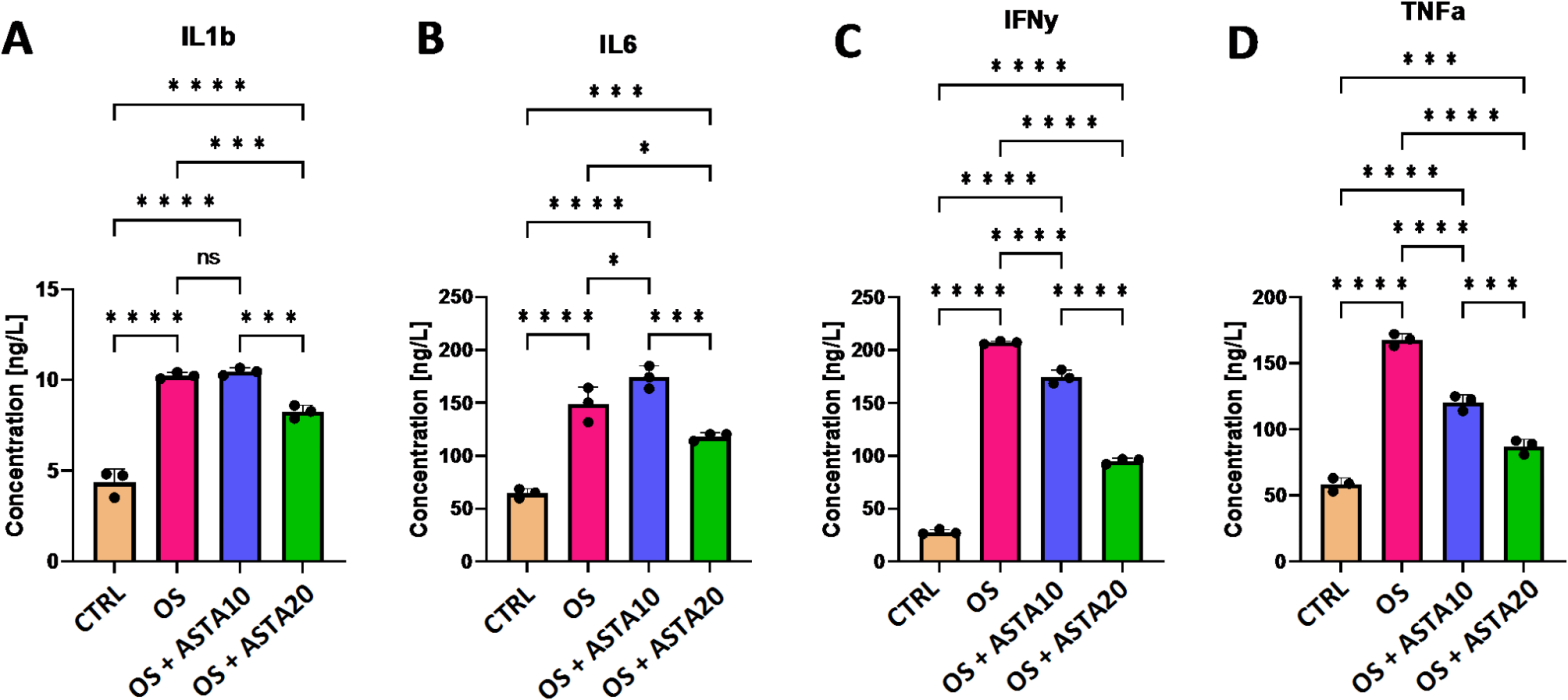
Effect of astaxanthin on the inflammatory response of horse PBMCs. (**A**–**D**) Analysis of the protein levels of selected pro-inflammatory cytokines (IL-1β, IL-6, INFy, TNFα) in control cells (CTRL), oxidative stress-induced cells (OS) and oxidative stress-induced cells treated with astaxanthin at 10 µM (OS + ASTA10) or 20 µM (OS + ASTA20) by ELISA. Mean and standard deviation are given. Statistical significance is depicted as; * p-value < 0.05, ** p-value < 0.01, *** p-value < 0.001, **** p-value < 0.0001; ns, not significant.

Incubation with H_2_O_2_ increased the protein expression levels of the pro-inflammatory cytokines IL-1β, IL-6, INFy and TNFα, indicating that horse PBMCs elicit an inflammatory response when stimulated with hydrogen peroxide (Fig. 4A–D). Interestingly, the addition of astaxanthin at 10 µM decreased the inflammatory response by reducing the protein levels of INFy and TNFα (Fig. 4C,D). Most importantly, a concentration-dependent effect was observed for astaxanthin regarding its anti-inflammatory properties, demonstrated by the decrease of protein level of all tested cytokines (IL-1β, IL-6, INFy and TNFα) significantly different between the treatment with 10 µM and 20 µM (Fig. 4A–D). Altogether, these results indicate the anti-inflammatory potential of astaxanthin in horse-derived PBMCs.

### The effect of astaxanthin oral supplementation in racehorses on the expression of genes associated with mitochondrial functionality and oxidative stress in PBMCs

The figures below (from Figs. 5, 6, 7, 8, 9, 10, 11, 12, 13, 14, 15, 16 and 17) represent the average relative gene expression (2^− ΔΔCt^) for the control and the astaxanthin supplemented groups at the three time points, together with the relative gene expression of all subjects individually.

**Fig. 5:**
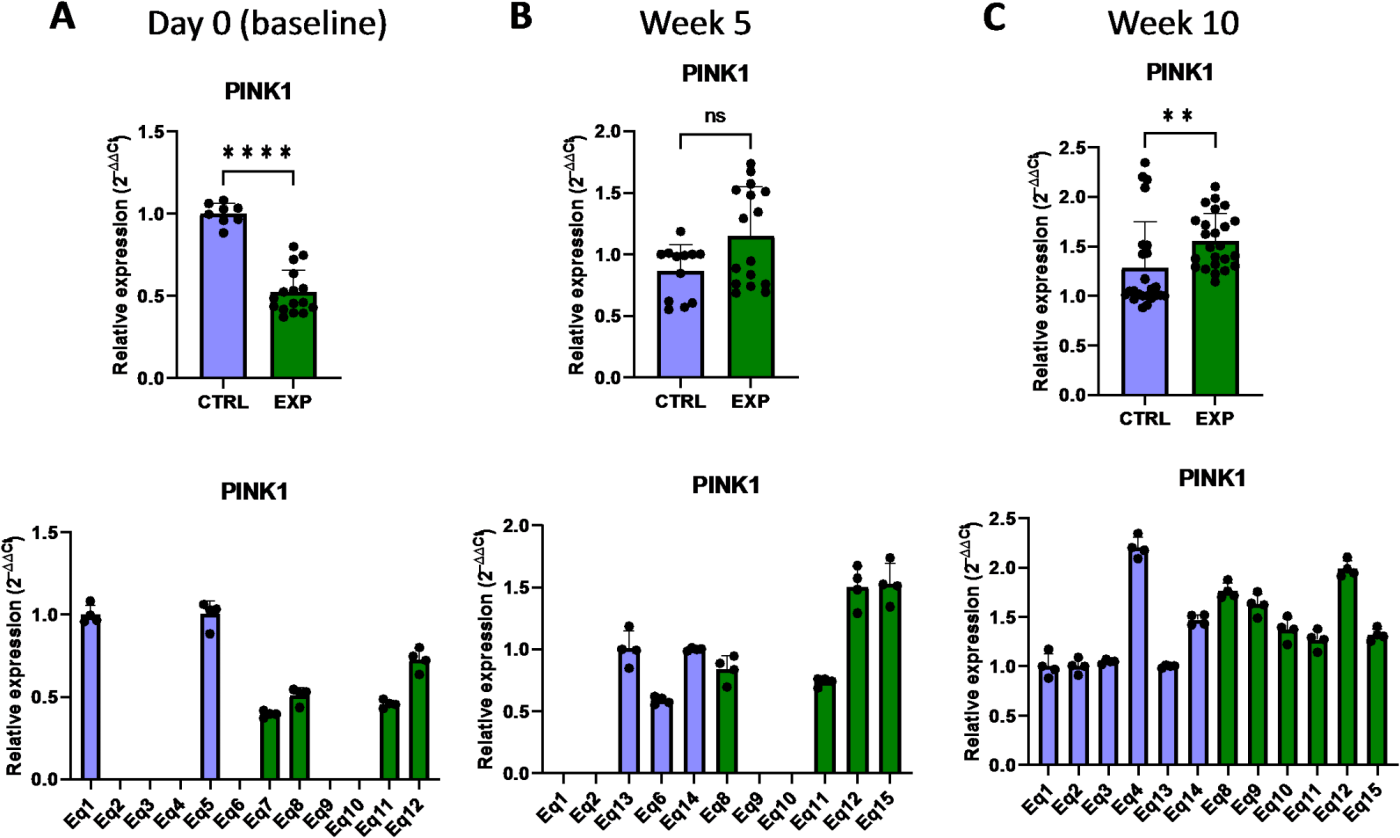
Effect of the oral astaxanthin supplementation in the *PINK1* gene expression of horse-derived PBMCs. Analysis of the *PINK1* gene expression levels by qRT-PCR at day 0 (**A**), week 5 (**B**) and week 10 (**C**) in horse-derived PBMCs. CTRL, non-treated control; EXP, supplemented group. Graphs in the top row represent the average of all subjects, and graphs in the bottom row represent the values for each subject individually. Mean and standard deviation are given. Statistical significance is depicted as; * p-value < 0.05, ** p-value < 0.01, *** p-value < 0.001, **** p-value < 0.0001; ns, not significant.

**Fig. 6:**
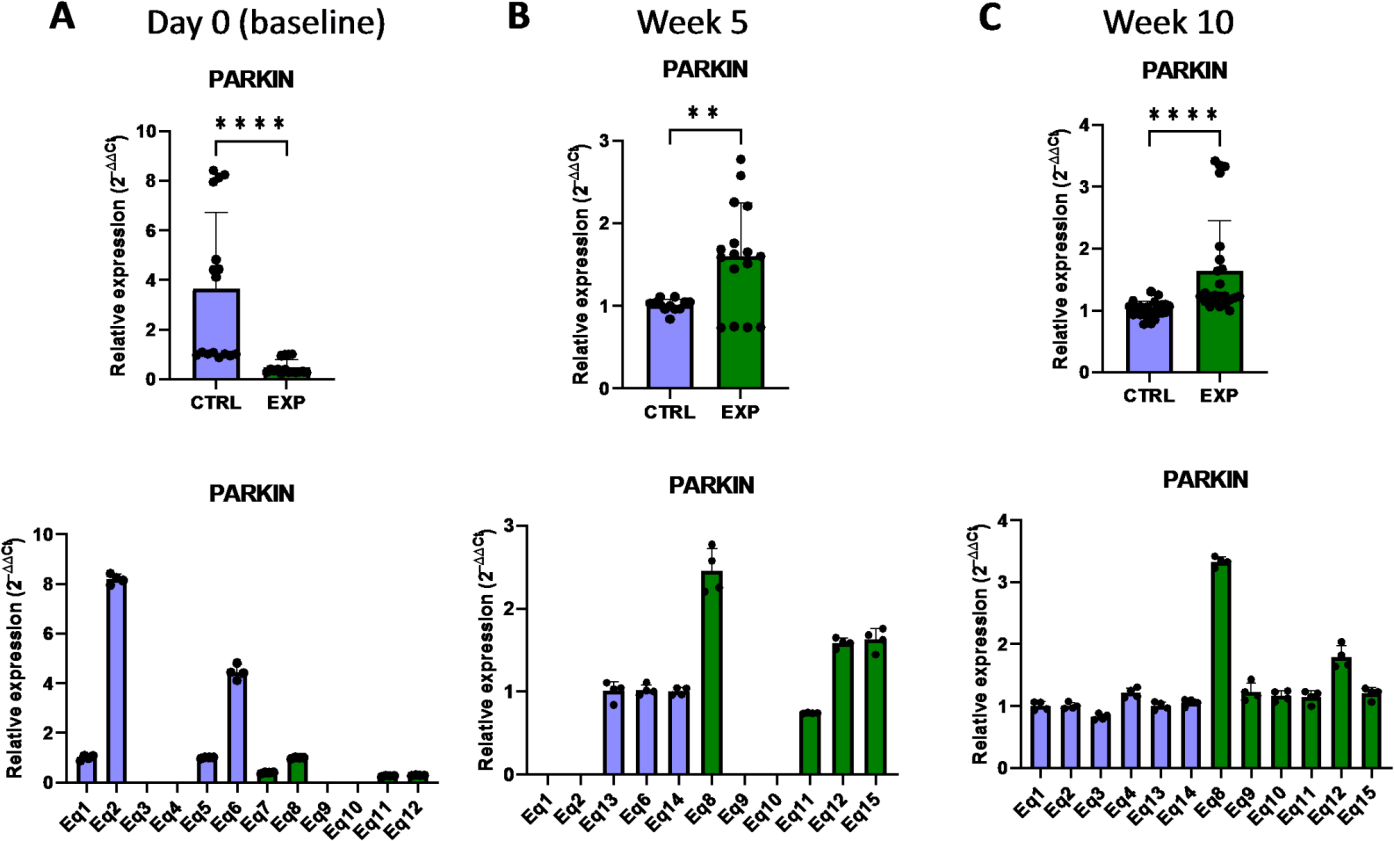
Effect of the oral astaxanthin supplementation in the *PARKIN* gene expression of horse-derived PBMCs. Analysis of the *PARKIN* gene expression levels by qRT-PCR at day 0 (**A**), week 5 (**B**) and week 10 (**C**) in horse-derived PBMCs. CTRL, non-treated control; EXP, supplemented group. Graphs in the top row represent the average of all subjects, and graphs in the bottom row represent the values for each subject individually. Mean and standard deviation are given. Statistical significance is depicted as; * p-value < 0.05, ** p-value < 0.01, *** p-value < 0.001, **** p-value < 0.0001; ns, not significant.

PINK1 (PTEN Induced Kinase 1) is a mitochondrial kinase directly related with the mitochondrial quality control, participating in the mitophagy process^16^. In damaged mitochondria, which are a source of reactive oxygen species that cause severe molecular damage to the cell, PINK1 is not properly internalized and binds to the outer mitochondrial membrane. Then, PINK1 recruits PARKIN (Parkin RBR E3 Ubiquitin Protein Ligase) protein and target damaged mitochondria for degradation through autophagy^16,17^. Removal of damaged mitochondria therefore helps to reduce the accumulation of ROS, to limit the ROS-derived cellular damage such as lipid peroxidation, and make a more efficient use of the cellular energy. The presented results showed that, although at day 0 (baseline) the expression level of *PINK1* and *PARKIN* was lower in the supplemented group compared to the control group, 10 weeks after the supplementation the expression of *PINK1* and *PARKIN* increased compared to the control group, indicating that oral supplementation with astaxanthin induced the expression of these genes in vivo (Figs. 5 and 6). The expression level of *PARKIN* was already increased compared to control after only 5 weeks of supplementation (Fig. 6), demonstrating that this gene presented a faster response compared to *PINK1.* Overall, oral intake of the astaxanthin supplement increased the expression of genes involved in the mitophagy process that actively protect from cellular oxidative stress.

**Fig. 7:**
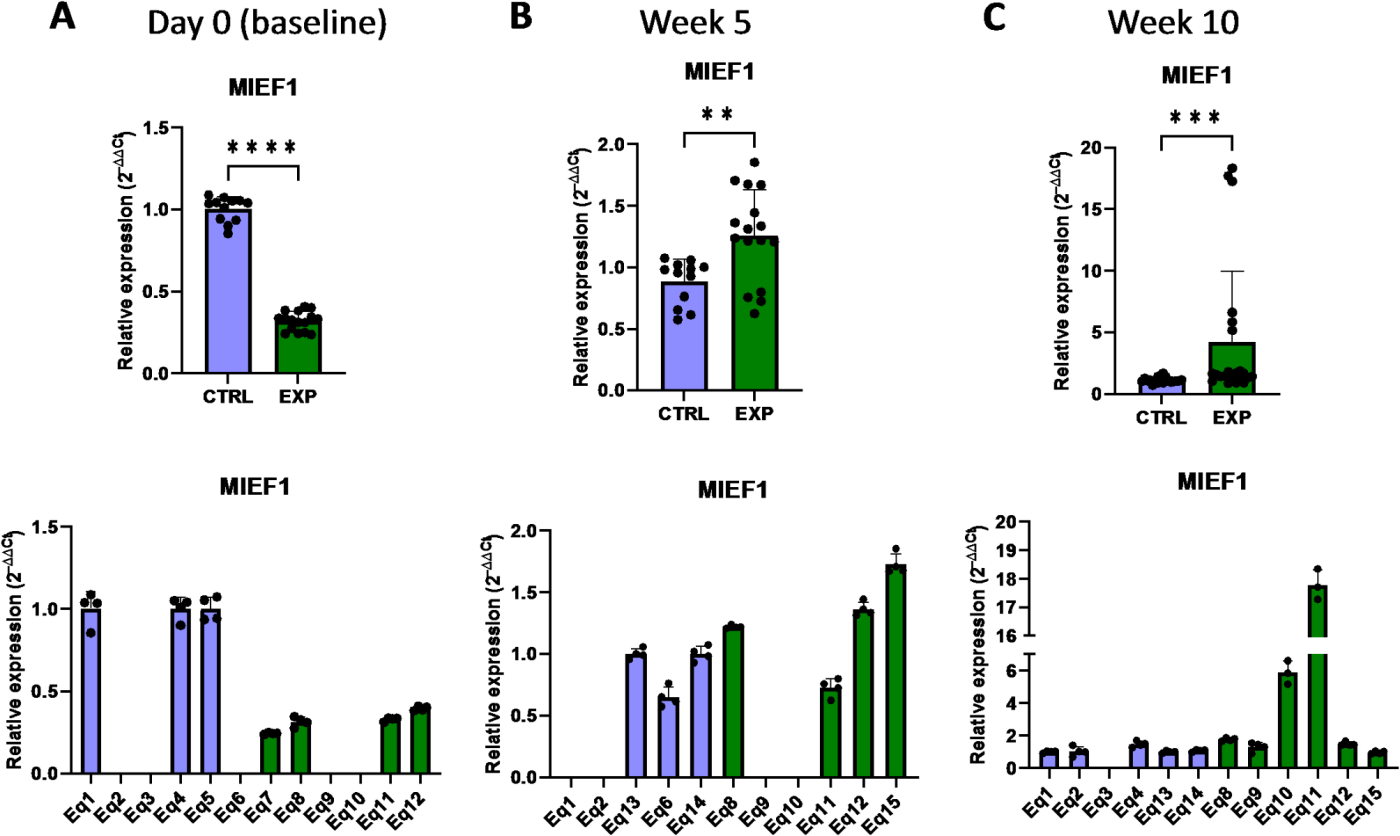
Effect of the oral astaxanthin supplementation on the *MIEF1* gene expression of horse-derived PBMCs. Analysis of the *MIEF1* gene expression levels by qRT-PCR at day 0 (**A**), week 5 (**B**) and week 10 (**C**) in horse-derived PBMCs. CTRL, non-treated control; EXP, supplemented group. Graphs in the top row represent the average of all subjects, and graphs in the bottom row represent the values for each subject individually. Mean and standard deviation are given. Statistical significance is depicted as; * p-value < 0.05, ** p-value < 0.01, *** p-value < 0.001, **** p-value < 0.0001; ns, not significant.

**Fig. 8:**
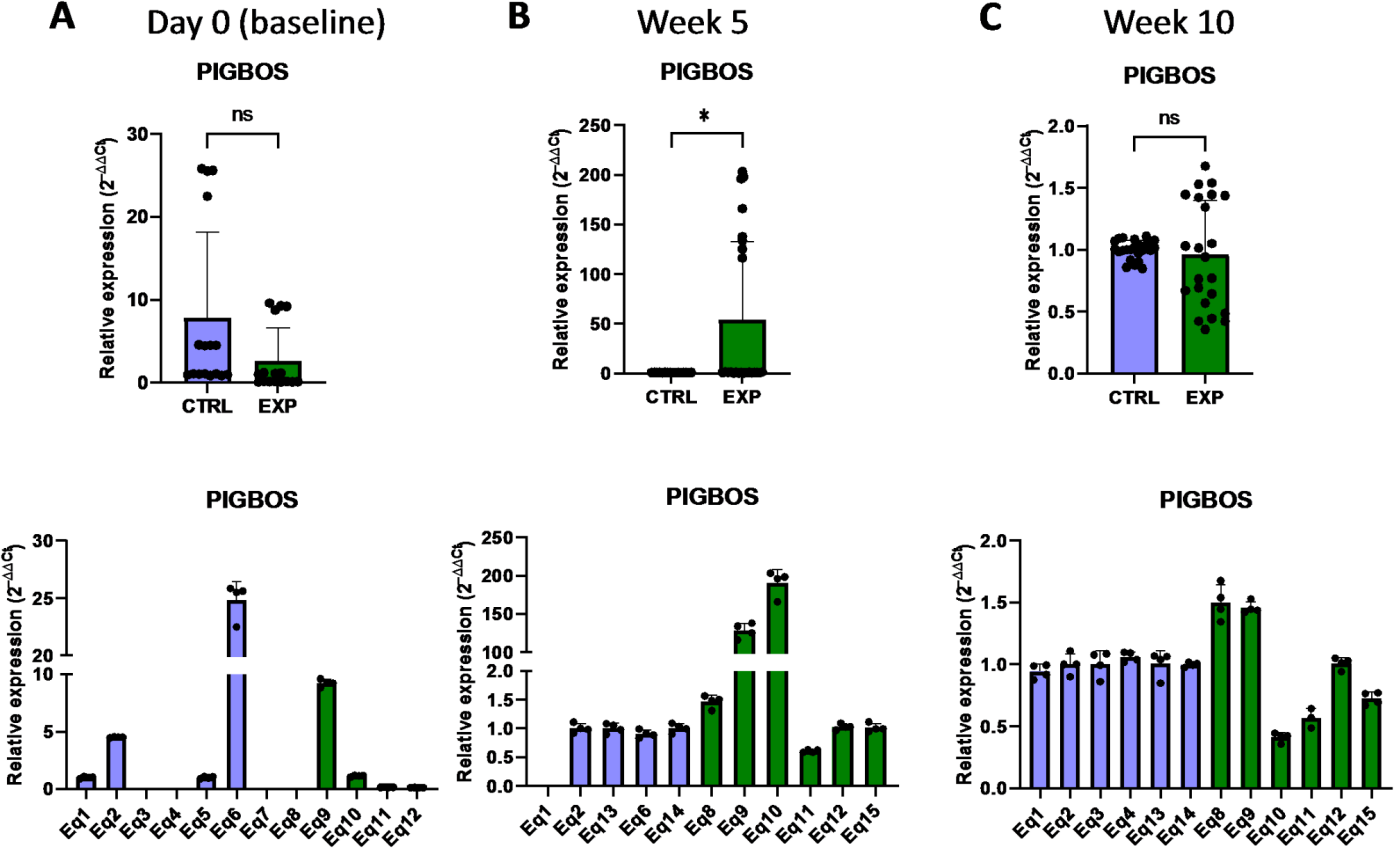
Effect of the oral astaxanthin supplementation on the *PIGBOS* gene expression of horse-derived PBMCs. Analysis of the *PIGBOS* gene expression levels by qRT-PCR at day 0 (**A**), week 5 (**B**) and week 10 (**C**) in horse-derived PBMCs. CTRL, non-treated control; EXP, supplemented group. Graphs in the top row represent the average of all subjects, and graphs in the bottom row represent the values for each subject individually. Mean and standard deviation are given. Statistical significance is depicted as; * p-value < 0.05, ** p-value < 0.01, *** p-value < 0.001, **** p-value < 0.0001; ns, not significant.

Further analyses indicate the impact of astaxanthin supplementation on the regulation of genes associated with mitochondrial dynamics and structure. *MIEF1* (mitochondrial elongation factor 1) encodes an outer mitochondrial membrane protein that participates in the mitochondrial membrane dynamics by regulating mitochondrial fission and fusion^18^. It has been shown that *MIEF1* deficiency impaired mitochondrial respiration and dynamics, leading to induced mitochondrial oxidative stress^19^. The results of the present study indicated that, initially, the *MIEF1* expression level was lower in the supplemented group than in the control group at day 0 (Fig. 7). However, the expression of the *MIEF1* gene was activated upon astaxanthin supplementation, being higher in the supplementad group after 5 weeks and 10 weeks (Fig. 7). The association of mitochondria and other cellular organelles are mediated by a number of proteins. Among them, *PIGBOS* (PIGB Opposite Strand) encodes for a microprotein that localizes to the mitochondrial outer membrane where it interacts with CLCC1, mediating in the unfolded protein response (UPR) originated by endoplasmic reticulum stress. It has been demonstrated that loss of PIGBOS leads to heightened UPR and increased cell death^20^. In this regard, the presented results demonstrated that the expression of *PIGBOS* increased in response to the oral supplementation with astaxanthin, evidenced by the increased mRNA levels of *PIGBOS* after 5 weeks compared to the control group (Fig. 8). Collectively, these results demonstrate that astaxanthin promotes the expression of genes that participate in the structure and function of mitochondria.

**Fig. 9:**
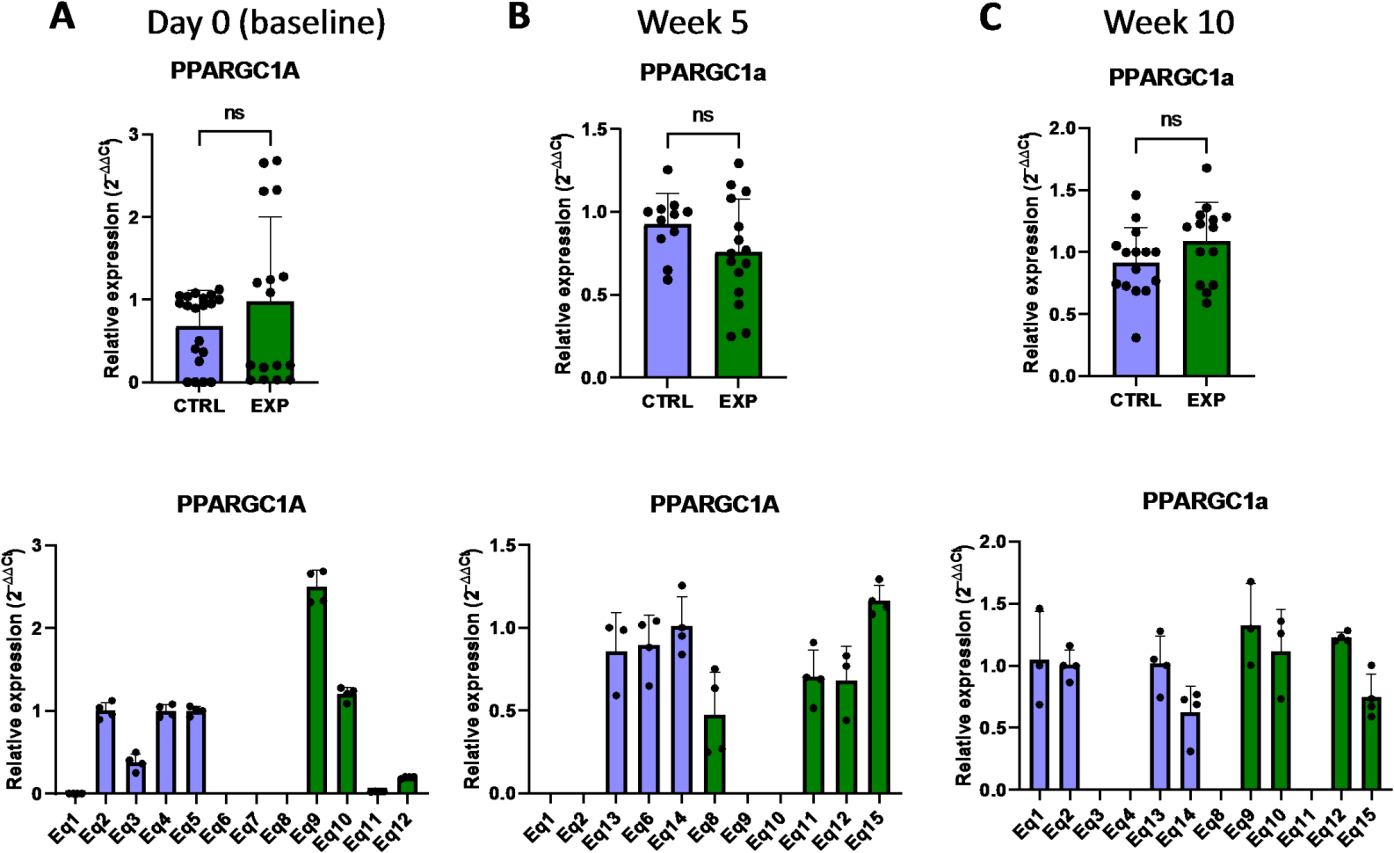
Effect of the oral astaxanthin supplementation on the *PPARGC1A* gene expression of horse-derived PBMCs. Analysis of the *PPARGC1A* gene expression levels by qRT-PCR at day 0 (**A**), week 5 (**B**) and week 10 (**C**) in horse-derived PBMCs. CTRL, non-treated control; EXP, supplemented group. Graphs in the top row represent the average of all subjects, and graphs in the bottom row represent the values for each subject individually. Mean and standard deviation are given. Statistical significance is depicted as; * p-value < 0.05, ** p-value < 0.01, *** p-value < 0.001, **** p-value < 0.0001; ns, not significant.

**Fig. 10:**
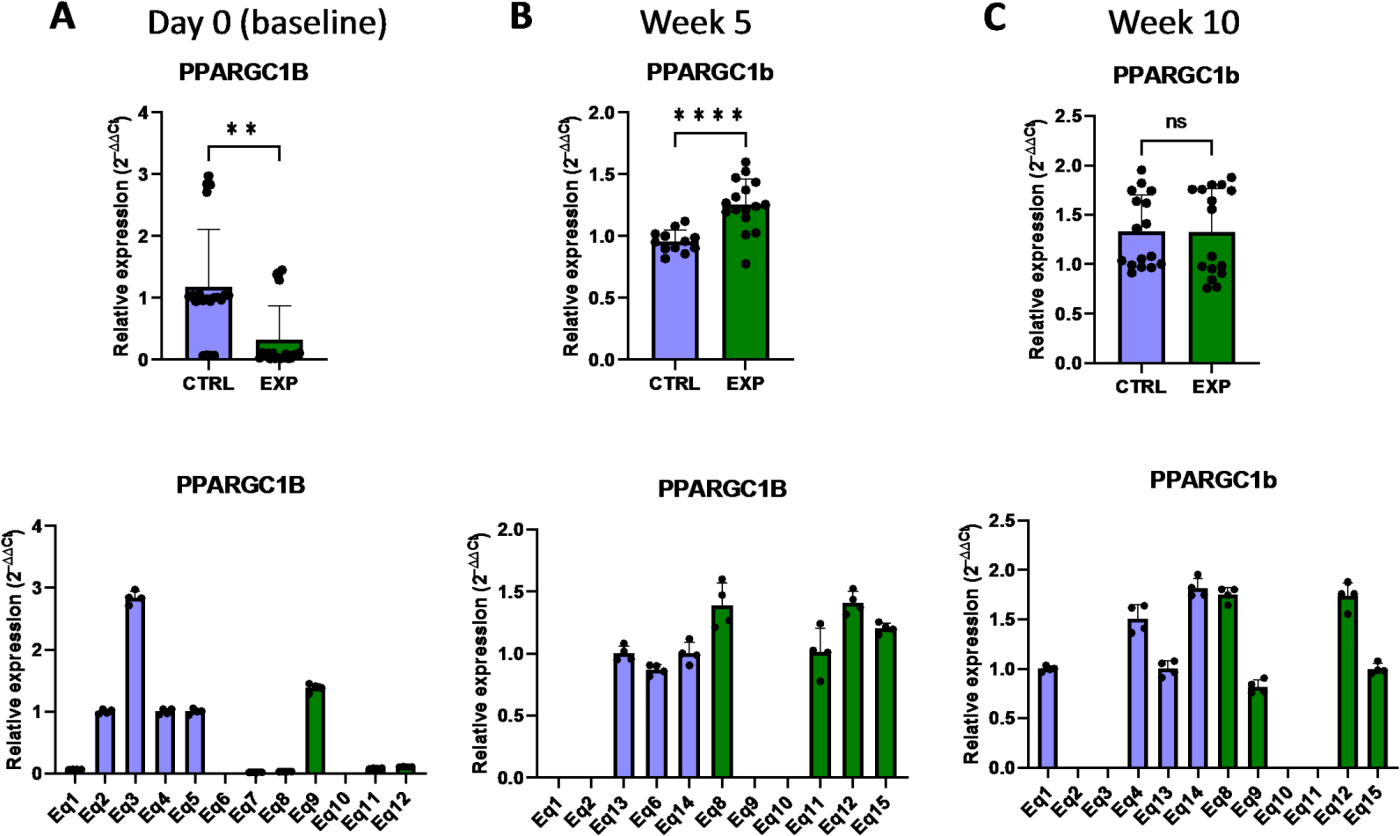
Effect of the oral astaxanthin supplementation on the *PPARGC1B* gene expression of horse-derived PBMCs. Analysis of the *PPARGC1B* gene expression levels by qRT-PCR at day 0 (**A**), week 5 (**B**) and week 10 (**C**) in horse-derived PBMCs. CTRL, non-treated control; EXP, supplemented group. Graphs in the top row represent the average of all subjects, and graphs in the bottom row represent the values for each subject individually. Mean and standard deviation are given. Statistical significance is depicted as; * p-value < 0.05, ** p-value < 0.01, *** p-value < 0.001, **** p-value < 0.0001; ns, not significant.

**Fig. 11:**
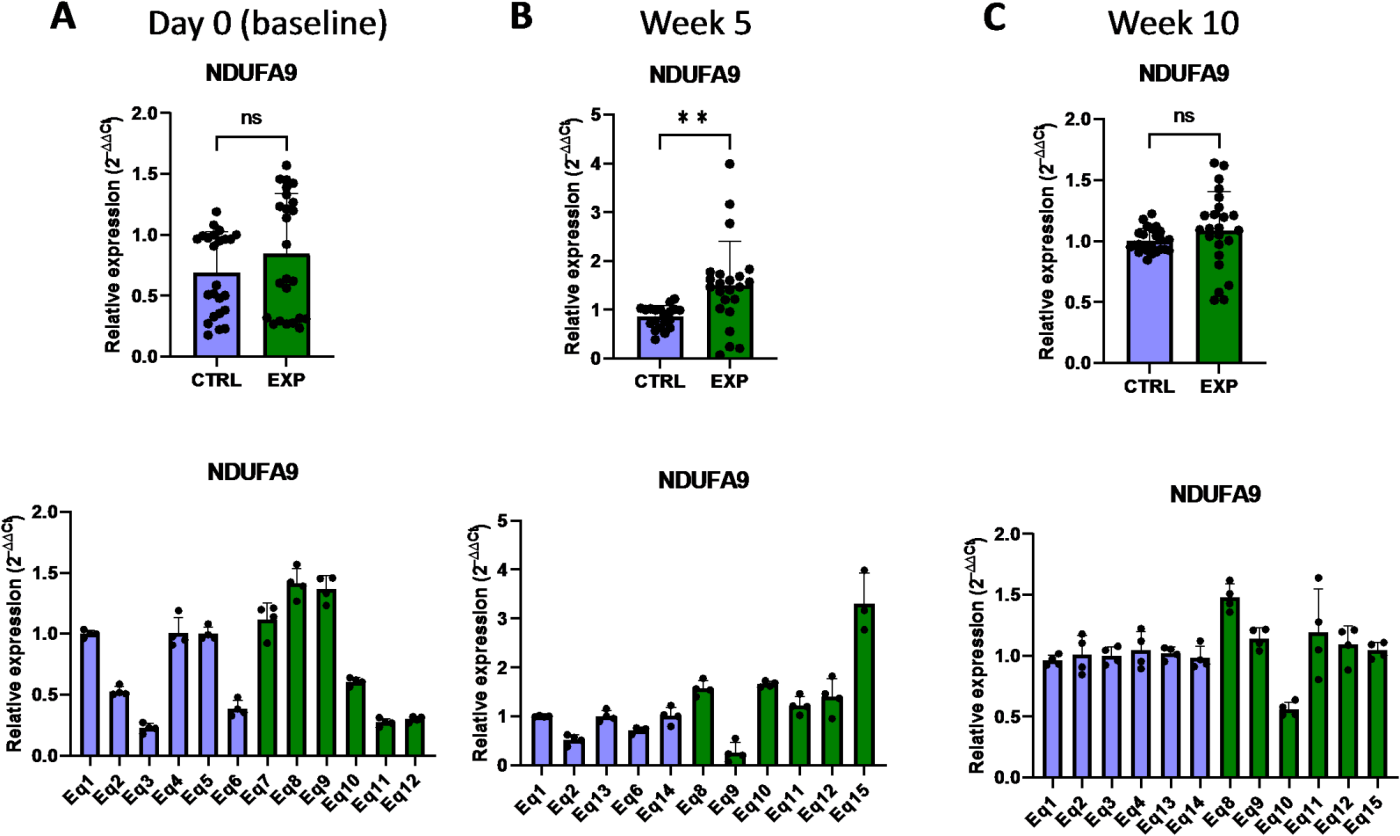
Effect of the oral astaxanthin supplementation on the *NDUFA9* gene expression of horse-derived PBMCs. Analysis of the *NDUFA9* gene expression levels by qRT-PCR at day 0 (**A**), week 5 (**B**) and week 10 (**C**) in horse-derived PBMCs. CTRL, non-treated control; EXP, supplemented group. Graphs in the top row represent the average of all subjects, and graphs in the bottom row represent the values for each subject individually. Mean and standard deviation are given. Statistical significance is depicted as; * p-value < 0.05, ** p-value < 0.01, *** p-value < 0.001, **** p-value < 0.0001; ns, not significant.

In order to evaluate whether the supplementation with astaxanthin regulates the expression of genes involved in the energy metabolism of the cell, some genes associated with the mitochondrial processes linked to ATP production we selected for further analyses. *PPARGC1A* and *PPARGC1B* (peroxisome proliferator-activated receptor gamma coactivator 1 alpha/beta) are transcriptional coactivators of the PPAR superfamily highly expressed in oxidative tissues, participating in the oxidative phosphorylation and fatty acid oxidation^21,22^. *PPARGC1A* is actively involved in reactive oxygen species detoxification by regulating the expression of mitochondrial antioxidant genes, such as manganese superoxide dismutase, catalase, peroxiredoxin 3 and 5, uncoupling protein 2, thioredoxin 2, and thioredoxin reductase^21,22^. *PPARGC1B* specifically enhances the mitochondrial activity and anabolic profile by the effect on the mitochondrial function and protection from oxidative stress^21^. The analysis of PBMCs isolated from supplemented horses revealed that the basal expression levels of *PPARGC1A* were similar in the control and supplemented group, and astaxanthin intake did not change this phenotype during the 10 weeks period (Fig. 9). However, *PPARGC1B* expression responded to the astaxanthin supplementation, since its expression levels were initially lower in the supplemented group at day 0 but increased at week 5, being higher than in the control group (Fig. 10). Mitochondria produce most of the cellular energy during the oxidative phosphorylation process. In this regard, *NDUFA9* (NADH: ubiquinone oxidoreductase subunit A9) encodes for a protein subunit of the complex I, and lack of a functional NDUFA9 has been associated with defects of the assembly and stability of this complex^23,24^. According to the results presented in this study, astaxanthin supplementation for 5 weeks increased the expression levels of *NDUFA9* compared to the control group (Fig. 11). Altogether, the results showed that supplementation with astaxanthin increases the expression of genes related to the energy production in mitochondria.

**Fig. 12:**
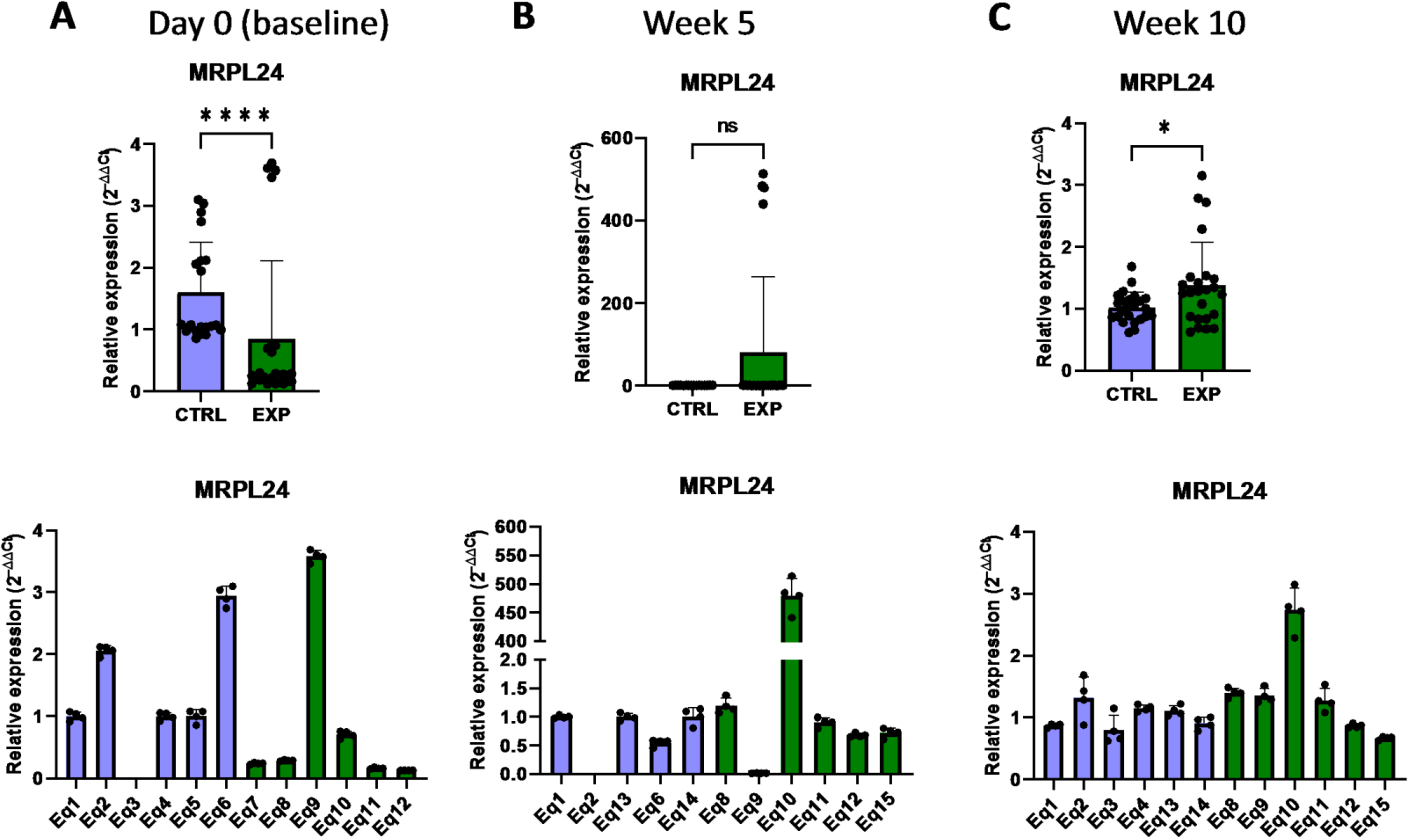
Effect of the oral astaxanthin supplementation on the *MRPL24* gene expression of horse-derived PBMCs. Analysis of the *MRPL24* gene expression levels by qRT-PCR at day 0 (**A**), week 5 (**B**) and week 10 (**C**) in horse-derived PBMCs. CTRL, non-treated control; EXP, supplemented group. Graphs in the top row represent the average of all subjects, and graphs in the bottom row represent the values for each subject individually. Mean and standard deviation are given. Statistical significance is depicted as; * p-value < 0.05, ** p-value < 0.01, *** p-value < 0.001, **** p-value < 0.0001; ns, not significant.

**Fig. 13:**
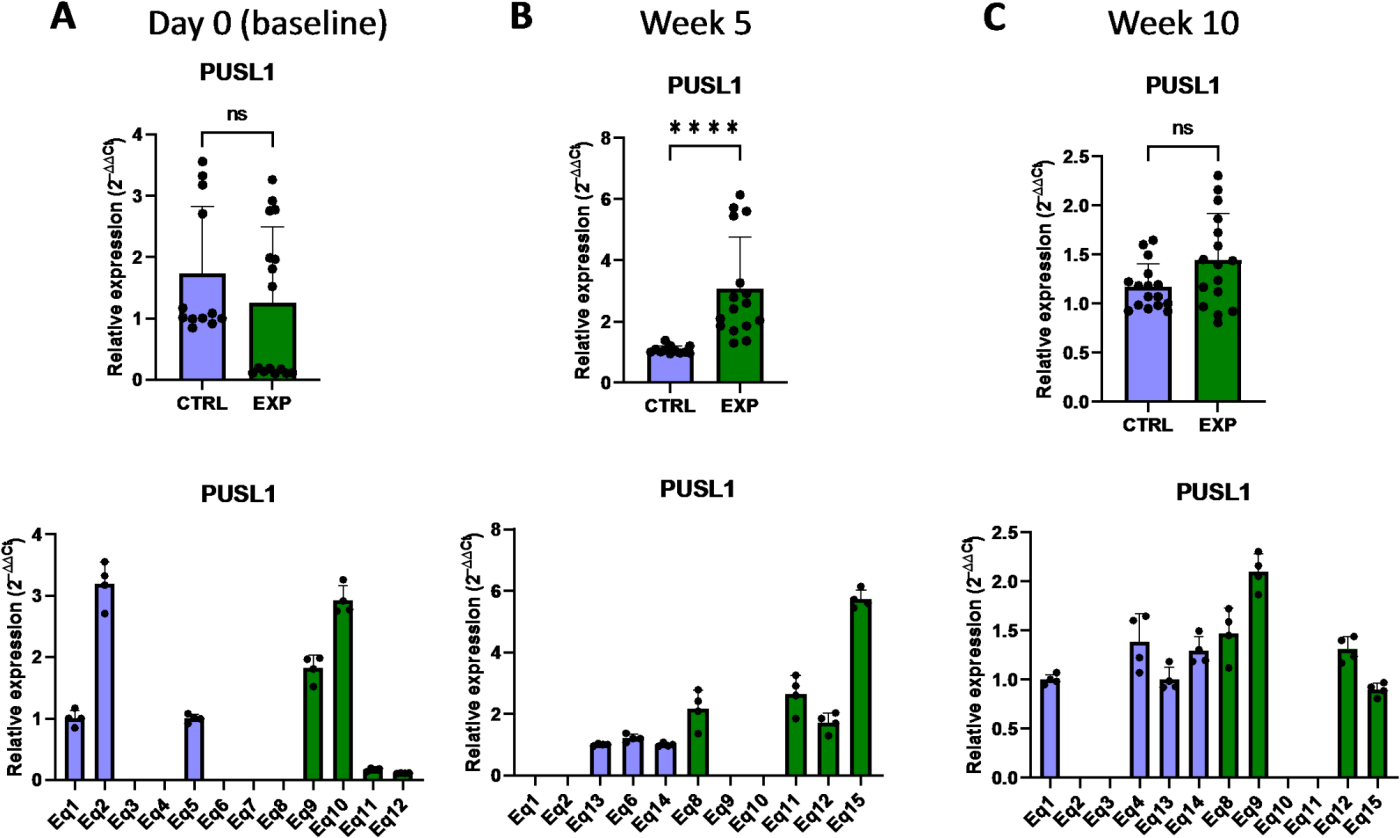
Effect of the oral astaxanthin supplementation on the *PUSL1* gene expression of horse-derived PBMCs. Analysis of the *PUSL1* gene expression levels by qRT-PCR at day 0 (**A**), week 5 (**B**) and week 10 (**C**) in horse-derived PBMCs. CTRL, non-treated control; EXP, supplemented group. Graphs in the top row represent the average of all subjects, and graphs in the bottom row represent the values for each subject individually. Mean and standard deviation are given. Statistical significance is depicted as; * p-value < 0.05, ** p-value < 0.01, *** p-value < 0.001, **** p-value < 0.0001; ns, not significant.

**Fig. 14:**
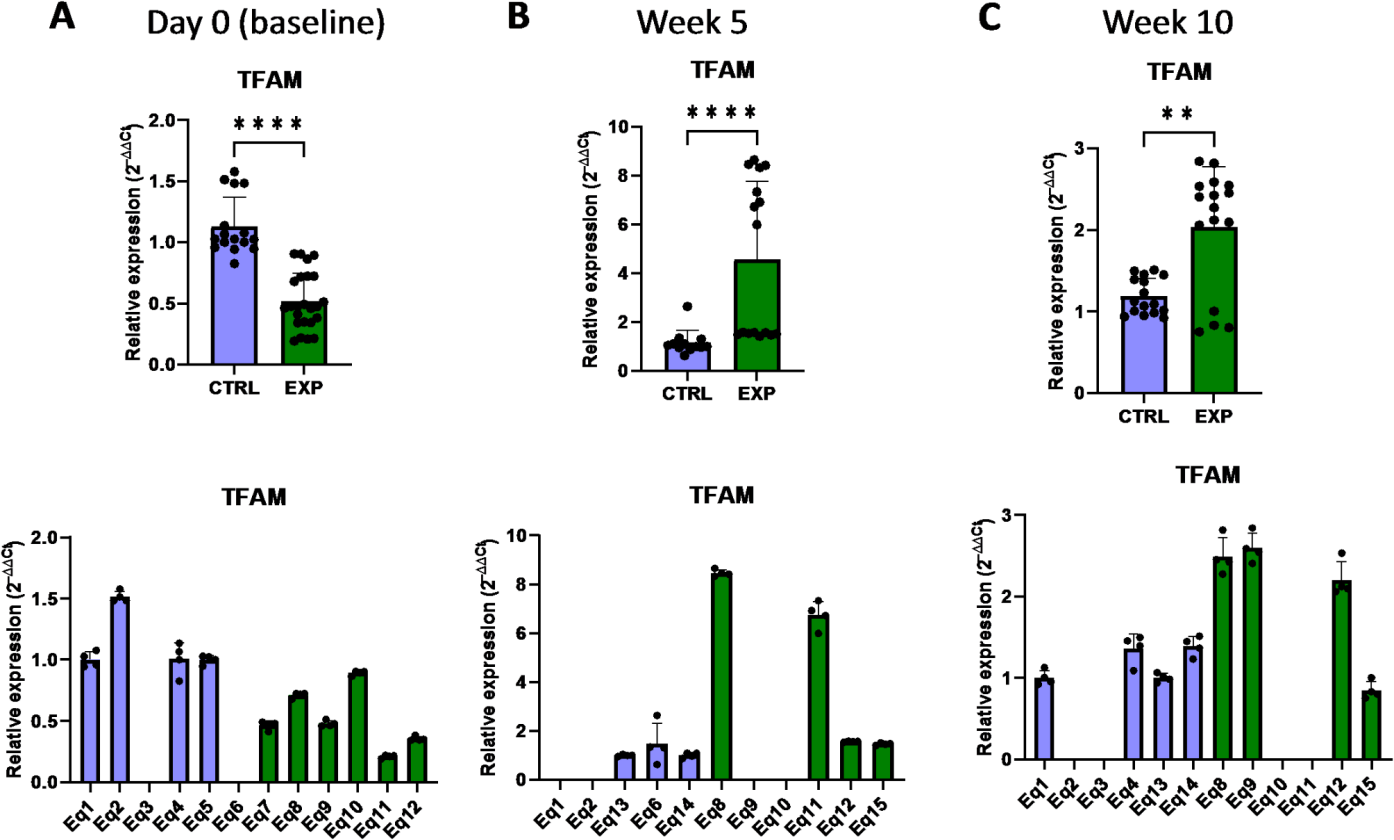
Effect of the oral astaxanthin supplementation on the *TFAM* gene expression of horse-derived PBMCs. Analysis of the *TFAM* gene expression levels by qRT-PCR at day 0 (**A**), week 5 (**B**) and week 10 (**C**) in horse-derived PBMCs. CTRL, non-treated control; EXP, supplemented group. Graphs in the top row represent the average of all subjects, and graphs in the bottom row represent the values for each subject individually. Mean and standard deviation are given. Statistical significance is depicted as; * p-value < 0.05, ** p-value < 0.01, *** p-value < 0.001, **** p-value < 0.0001; ns, not significant.

Mitochondrial DNA (mtDNA) encodes for proteins and enzymes directly related to the mitochondrial function and structure. The alteration of the mitochondrial gene expression machinery might lead to an impaired mitochondrial function and generate lethal levels of reactive oxygen species. In this regard, a key structural component of mitochondria is MRPL24 (mitochondrial ribosomal protein L24), the mitochondrial ribosomal protein large 24, which is 1 of the 82 protein components of mitochondrial ribosomes, playing an essential role in the mitochondrial translation process^25^. Indeed, it has been shown that absence or deficiency of this family of proteins may cause primary oxidative phosphorylation disorders^25^. Interestingly, the expression level of *MRPL24* gene was lower in supplemented group at baseline compared to the control group, but this phenotype changed to a higher expression in the astaxanthin-supplemented group after 10 weeks (Fig. 12). Another important structural and functional component of mitochondria is PUSL1 (tRNA pseudouridine synthase-like 1), a mitochondrial matrix protein and interacts with the mitoribosome, being required for the translation of transcripts within mitochondria^26^. These transcripts include key membrane proteins of the oxidative phosphorylation (OXPHOS) machinery located in the mitochondrial inner membrane, whose deficiency or malfunction may generate reactive oxygen species within mitochondria. Closely related to PUSL1, the transcriptional regulator TFAM (mitochondrial transcription factor A) is required for protein transcription, replication and packaging of mtDNA^27^. In this case, supplementation with astaxanthin increased the *PUSL1* and *TFAM* expression levels after 5 weeks compared to the control group (Figs. 13 and 14). Indeed, in the case of *TFAM*, this increase in expression levels was extended up to 10 weeks, despite initially (at baseline) lower levels of expression than in the control group (Fig. 14).

**Fig. 15:**
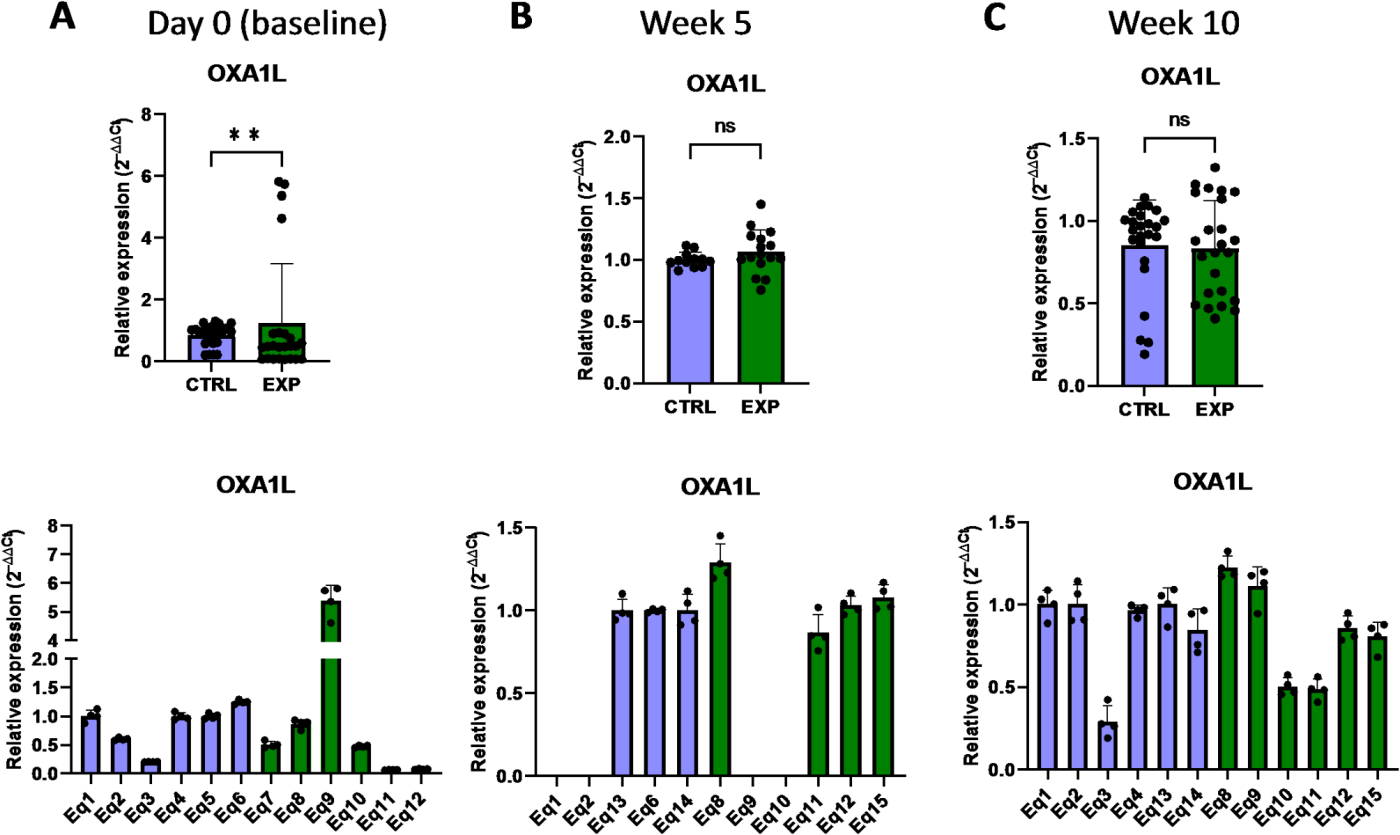
Effect of the oral astaxanthin supplementation on the *OXA1L* gene expression of horse-derived PBMCs. Analysis of the *OXA1L* gene expression levels by qRT-PCR at day 0 (**A**), week 5 (**B**) and week 10 (**C**) in horse-derived PBMCs. CTRL, non-treated control; EXP, supplemented group. Graphs in the top row represent the average of all subjects, and graphs in the bottom row represent the values for each subject individually. Mean and standard deviation are given. Statistical significance is depicted as; * p-value < 0.05, ** p-value < 0.01, *** p-value < 0.001, **** p-value < 0.0001; ns, not significant.

**Fig. 16:**
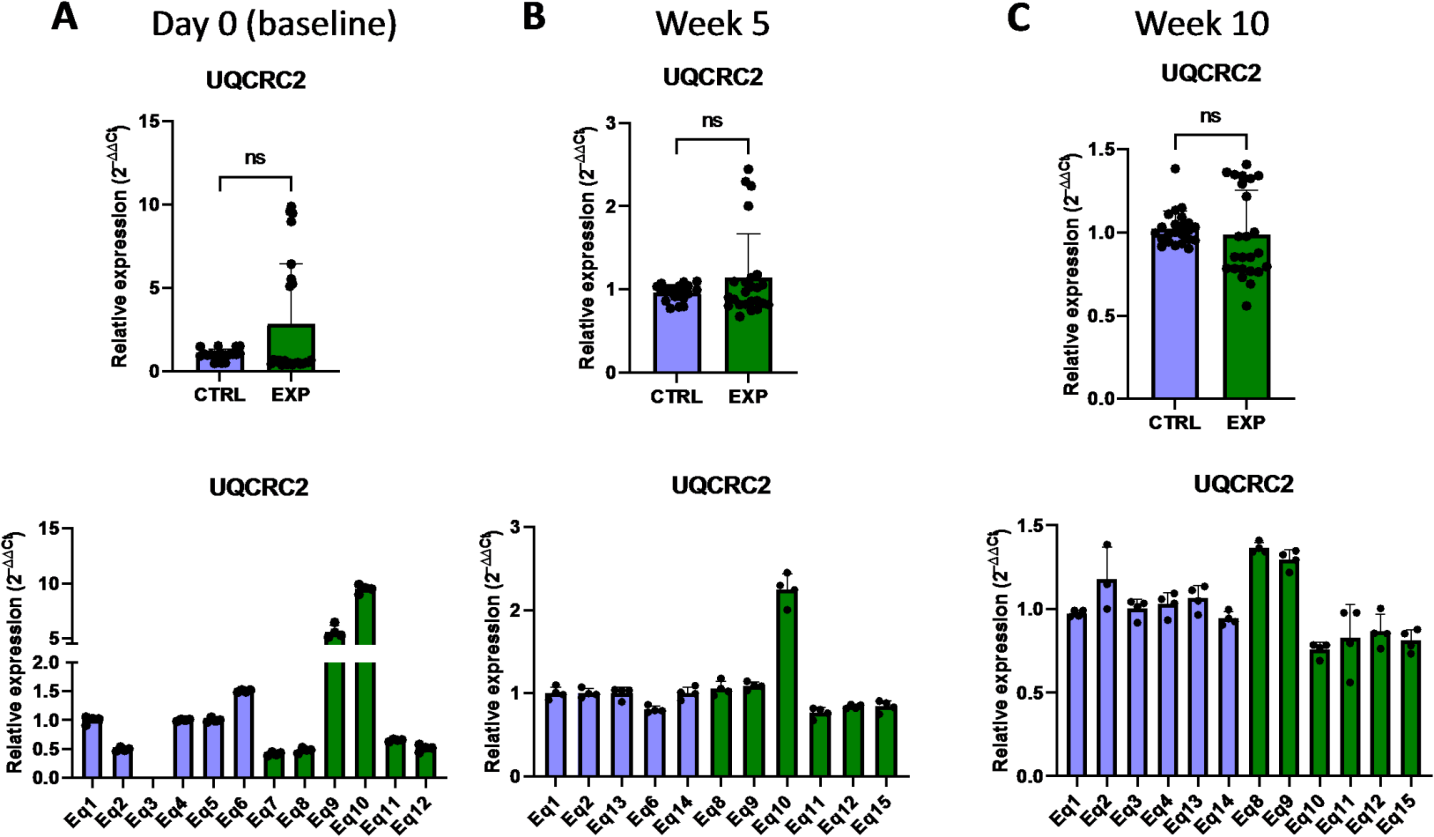
Effect of the oral astaxanthin supplementation on the *UQCRC2* gene expression of horse-derived PBMCs. Analysis of the *UQCRC2* gene expression levels by qRT-PCR at day 0 (**A**), week 5 (**B**) and week 10 (**C**) in horse-derived PBMCs. CTRL, non-treated control; EXP, supplemented group. Graphs in the top row represent the average of all subjects, and graphs in the bottom row represent the values for each subject individually. Mean and standard deviation are given. Statistical significance is depicted as; * p-value < 0.05, ** p-value < 0.01, *** p-value < 0.001, **** p-value < 0.0001; ns, not significant.

**Fig. 17:**
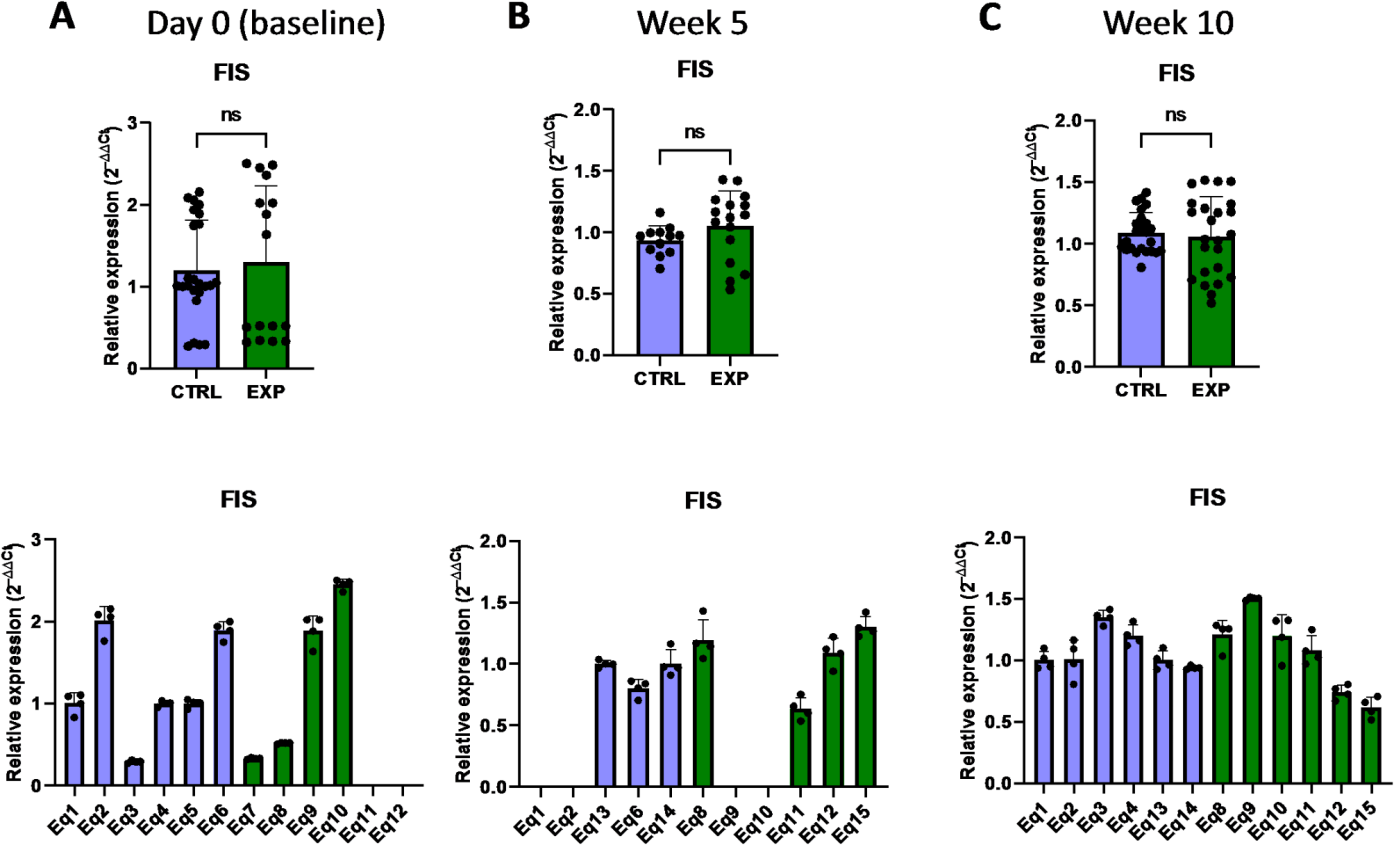
Effect of the oral astaxanthin supplementation on the *FIS* gene expression of horse-derived PBMCs. Analysis of the *FIS* gene expression levels by qRT-PCR at day 0 (**A**), week 5 (**B**) and week 10 (**C**) in horse-derived PBMCs. CTRL, non-treated control; EXP, supplemented group. Graphs in the top row represent the average of all subjects, and graphs in the bottom row represent the values for each subject individually. Mean and standard deviation are given. Statistical significance is depicted as; * p-value < 0.05, ** p-value < 0.01, *** p-value < 0.001, **** p-value < 0.0001; ns, not significant.

The expression of other genes associated with the protein expression within mitochondria, such as *OXA1L* (mitochondrial Inner Membrane Protein OXA1L) and *UQCRC2* (ubiquinol-cytochrome C reductase core protein 2)^28,29^, was not affected by astaxanthin supplementation (Figs. 15 and 16). In addition, no changes were observed in the expression of the FIS gene, involved in the mitochondrial fission (Fig. 17). Overall, these results revealed that the expression of genes involved in the metabolism of mtDNA is promoted in vivo upon supplementation with astaxanthin.

## Discussion

In horses, oxidative stress is a common issue that can lead to various health problems, including inflammation and compromised cellular functions. The present study aimed to evaluate the protective effects of astaxanthin against oxidative stress in horse-derived peripheral blood mononuclear cells (PBMCs) and to explore its potential anti-inflammatory properties and the effects on mitochondrial function. It has been demonstrated that astaxanthin exerts a positive effect on reducing the number of ROS-positive cells that were induced by oxidative stress. The application in vitro of hydrogen peroxide (H₂O₂) significantly increased the number of ROS-positive cells in horse-derived PBMCs, indicating induced oxidative stress. However, the addition of astaxanthin at the concentrations of 10 µM (OS + ASTA10) and 20 µM (OS + ASTA20) partially restored the cellular phenotype, as shown by a reduction in the number of ROS-positive cells and an increase in ROS-negative cells. Although these changes were not statistically significant, they suggest a trend towards the antioxidative effect of astaxanthin. This observation aligns with previous research demonstrating the antioxidant capacity of astaxanthin in various cell types, including its ability to scavenge free radicals and reduce oxidative damage^5,30^. Furthermore, the quantification of reactive nitrogen species (RNS) revealed an interesting phenomenon where astaxanthin increased the levels of cellular RNS, particularly in the alive cell population. This unexpected result suggests a complex interaction between astaxanthin and nitrosative stress pathways. The differential impact on RNS and ROS indicates that while astaxanthin can mitigate oxidative stress, it might also modulate nitrosative pathways, possibly through a feedback mechanism that needs further exploration^31^. Moreover, daily astaxanthin supplementation for 5 and 10 weeks increased the expression of *NRF1*, *SOD2*, and *GPX*, suggesting its role in priming cells for enhanced antioxidative responses. This is consistent with findings from other studies where astaxanthin supplementation upregulated antioxidant genes and enzymes, thus providing cellular protection against oxidative stress^32^.

The results proved also the anti-inflammatory potential of astaxanthin, related to its antioxidative properties. In vitro treatment of horse-derived PBMCs with H₂O₂ increased the protein expression of pro-inflammatory cytokines IL-1β, IL-6, IFNγ, and TNFα, indicating an inflammatory response and elevated oxidative stress levels. This is consistent with the well-documented phenomenon where oxidative stress can trigger inflammatory pathways, leading to the activation of immune cells and the release of pro-inflammatory cytokines. The addition of astaxanthin, particularly at 20 µM, significantly reduced the levels of these cytokines in a concentration-dependent manner. This decrease in cytokine levels suggests that astaxanthin effectively mitigates both oxidative stress and the subsequent inflammatory response. The antioxidant action of astaxanthin likely interrupts the feedback loop between oxidative stress and inflammation, thereby reducing the overall inflammatory burden. These findings are supported by previous studies that have shown the anti-inflammatory effects of astaxanthin, including the inhibition of pro-inflammatory cytokine production and suppression of NF-κB signaling pathways. For instance, Kim et al. (2011) reported that astaxanthin protects human retinal pigment epithelial cells from oxidative damage and inflammatory responses by inhibiting NF-κB activation^33^. Similarly, Park et al. (2010) demonstrated that astaxanthin supplementation decreased oxidative stress and inflammation in humans^34^, highlighting its potential as a therapeutic agent for inflammatory conditions.

The in vivo trial with horses further corroborated the in vitro findings. Our study focused on astaxanthin supplementation as a clinical treatment for influencing oxidative stress and inflammation, chosen over other approaches such as photobiomodulation, electrical stimulation, and ultrasound treatment, which may offer different and/or complementary effects. Oral supplementation with astaxanthin led to increased expression of genes associated with mitochondrial functionality and oxidative stress mitigation. Specifically, the expression of *PINK1* and *PARKIN*, genes involved in the mitophagy process, was elevated, indicating enhanced mitochondrial quality control mechanisms. This is particularly important for racehorses, as efficient mitophagy helps maintain optimal cellular function and energy production under the high metabolic demands of intensive physical activity. Mitophagy, the selective degradation of damaged mitochondria by autophagy, is crucial in preserving mitochondrial health and preventing cellular dysfunction. Mitophagy study is widely conducted through gene expression analysis, which provides valuable insights into the regulatory mechanisms governing this process^35,36^. In the context of high-performance racehorses, maintaining robust mitochondrial function is vital for sustaining stamina and performance. The presented findings that astaxanthin supplementation enhances mitophagy through the upregulation of *PINK1* and *PARKIN* genes suggest that astaxanthin could play a significant role in improving mitochondrial quality and overall cellular health in racehorses. This aligns with studies showing that astaxanthin promotes mitophagy and protects cells from oxidative stress-induced mitochondrial damage^37^. Additionally, astaxanthin enhanced the expression of genes linked to mitochondrial dynamics (*MIEF1* and *PIGBOS*) and energy metabolism (*PPARGC1B*, *NDUFA9*). The increased expression of these genes suggests that astaxanthin supplementation enhances mitochondrial structure, function, and bioenergetics, thus supporting cellular health and reducing oxidative damage. This is supported by previous research, which has demonstrated that improved mitochondrial dynamics and energy metabolism are associated with better cellular health and reduced oxidative stress^38^. Since the detailed characterization of mitophagy and mitochondrial dynamics, as well as gender-based differences, were beyond the scope of this study, future research performed with a different study design will be crucial to further elucidate these aspects. Importantly, while our findings in Arabian racehorses align with those in other large mammals^7,8,34,39^, species-specific differences in metabolism, immune responses, and oxidative stress susceptibility may influence astaxanthin’s effects. Monogastric species like horses and dogs absorb astaxanthin likely differently than ruminants, where fermentation might alter its bioavailability. Additionally, oxidative stress in racehorses is exercise-induced, whereas in cattle, it is linked to metabolic disorders. Altogether, although horses share some physiological and metabolic characteristics with other mammals, significant differences exist, especially in training responses and disease susceptibility, hence, results from equine studies cannot be directly extrapolated to humans.

Overall, the findings presented in this study indicate that astaxanthin has significant potential as an antioxidant and anti-inflammatory agent in horse-derived PBMCs. The in vivo data further support its role in enhancing mitochondrial function and protecting against oxidative stress. These results suggest that astaxanthin could be a valuable dietary supplement for equine health, promoting resilience against oxidative and inflammatory challenges.

## Materials and methods

### Horses and blood sampling

The in vivo part of this study included 12 (7 stallions and 5 mares) privately owned 3-year-old Arabian horses in regular training for flat races at Służewiec Race Track in Warsaw. All horses were from the same training stable, were trained by one trainer and maintained similar training level during the study. They were kept in standard stalls, fed on a standard diet for racing Arabians, including hay, oats, and concentrate balanced to meet the nutritional recommendations. Salt and water were available *ad libitum*. The horses were clinically healthy, as confirmed by a certified vet surgeon; dewormed and vaccinated according to the routine schedule, not earlier than 3 weeks before the onset of the study. In the case of concomitant disabilities or diseases, horses were excluded from the study.

At the beginning of the study (in April) the horses were randomly allocated in the supplemented group (4 stallions and 2 mares) and control group (3 stallions and 3 mares). The supplemented group received daily astaxanthin supplementation, orally, at a dose of 250 mg per horse which corresponded to the dose of 0.52–0.58 mg/kg body weight. The control group received placebo (physiological saline). Stallions and mares trained together in a mixed group with the same intensity at the same place (Służewiec Racetrack in Warsaw), according to the exercise schedule designed by the trainer.

Blood samples were collected monthly (week 0 – before supplementation and during supplementation in week 5 and week 10) as a part of health examination, in the morning, before any activity, from the jugular vein. During the sampling procedure, the horses were handled by their regular riders to minimize the stress, as recommended by the Ethical Committee guidelines. All the procedures of blood sampling were performed as part of routine health examination, therefore, according to the European directive EU/2010/63 and Polish regulations regarding experiments on animals, there was no need for the approval of the Ethics Committee for the described procedures, which qualified as non-experimental clinical veterinary practices, and excluded from the directive. A written consent for the use of blood for scientific analyses was obtained from the trainer.

## Isolation and culture of horse peripheral blood mononuclear cells (PBMC)

Fresh blood from horses was collected into heparin tubes. PBMCs isolation was performed as described previously^40^. Briefly, PBMCs were isolated by density gradient centrifugation for 30 min at 400 g, at room temperature (MPW-352R, MPW Med. Instruments, Warsaw, Poland), using Histopaque-1077 (Sigma-Aldrich/Merck, Poznan, Poland). A buffy coat layer of PBMCs cells was collected and washed three times with Dulbecco’s Phosphate Buffered Saline (DPBS, Merck, Poznan, Poland). Cells were cultured on cell culture flask 25 cm^2^ in RPMI 1640 medium (Sigma-Aldrich/Merck, Poznan, Poland) supplemented with 10% fetal bovine serum (Sigma-Aldrich/Merck, Poznan, Poland) and 1% penicillin-streptomycin antibiotic (PS, Biowest). Cells were incubated at 37 °C, 5% CO_2_.

## Induction of oxidative stress in PBMCs

PBMCs were suspended at a density of 1 × 10^6^ cells/ml and treated with hydrogen peroxide (H_2_O_2_) at a concentration of 100 µM in culture medium without FBS to induce cellular oxidative stress, as previously described^41^. After 6 hours, cells were treated with astaxanthin at 10 µg/ml and 20 µg/ml for 24 h^4^. Then, the expression of selected markers at gene and protein level, the oxidative stress and the nitrosative stress were assessed as described below.

### MUSE analysis of oxidative stress and nitrosative stress of PBMCs

Oxidative stress was analyzed using the Muse Oxidative Stress Kit (Luminex/Merck, Poznan, Poland) and nitric oxide levels using MUSE Nitric Oxide Kit (Luminex/Merck, Poznan, Poland)^42^. Analysis was performed according to the protocol from the manufacturer. Readings were taken using a MUSE Cell Analyser (Sigma-Aldrich/Merck, Poznan, Poland).

## Quantitative Real-Time reverse transcription polymerase chain reaction (RT-qPCR)

Total RNA was isolated from PBMCs following the previously described method of Chomczynski and Sacchi^43^. Blood was collected into the Tempus™ Blood RNA Tubes (Thermo Fisher Scientific, Warsaw, Poland) and poured into a 50 ml falcon tube. The total volume was brought to 12 ml by the addition of PBS (Merck, Poznan, Poland). The tubes were vortexed vigorously at maximum vortex speed for 30 s. The samples were then centrifuged at 4 °C at 4000xg for 30 min (MPW-352R, MPW Med. Instruments, Warsaw, Poland). The pellet was suspended in 400 µl RNA Wash Buffer 1 (BLIRT S.A, Gdansk, Poland) and then the total RNA was isolated using Extractme Total RNA Kit (BLIRT S.A, Gdansk, Poland) according to the manufacturer’s instructions. The purity and quantity of RNA was assessed at 260 and 280 nm with a spectrophotometer (Epoch, Biotek, Bad Friedrichshall, Germany). 300 ng of total RNA was treated with DNase I (Thermo Fisher Scientific, Warsaw, Poland) to remove genomic DNA. cDNA was then synthesized on the RNA template using RevertAid RT (Thermo Fisher Scientific, Warsaw, Poland) according to the manufacturer’s protocols. Digestion of genomic DNA and cDNA synthesis were performed using a T100 Thermal Cycler (Bio-Rad, Hercules, CA, USA). The obtained cDNA was then used for quantitative PCR. Gene expression levels were evaluated using SensiFAST SYBR Green Kit (Bioline, London, UK)^44^. 5 µL of SensiFAST SYBR Master mix, 2.5 µL of targeted primer and 2.5 µL of tested cDNA were added to each reaction. All RT-qPCR reactions were performed using CFX Connect Real-Time PCR Detection System (Bio-Rad, Hercules, CA, USA) and BioRad CFX Maestro software. Thermal cycle conditions were as follows: 95 °C for 2 min, then 40 cycles at 95 °C for 15 s, annealing for 15 s in temperature specified for tested primers, and elongation at 72 °C for 15 s. The transcript levels were normalized to glyceraldehyde-3-phosphate dehydrogenase (*Gapdh*) as a control and calculated using the 2^−ΔΔCQ^ method. The sequences for all used primers are listed in Table 1.

## Analysis of protein levels using ELISA assays (IL1β, IL6, IFNy, TNFα)

Protein levels of IL1β, IL6, IFNy and TNFα were analyzed in culture media using enzyme linked immunosorbent assays (ELISA) as previously described^40^. For this purpose, the Horse Interleukin 1 Beta ELISA Kit, Horse Interleukin 6 ELISA Kit, Horse Interferon γ ELISA Kit and Horse Tumour Necrosis Factor Alpha ELISA Kit (BT LAB) were used. Analyses were performed according to the manufacturer’s protocols and readings were taken at 450 nm using a plate reader (Epoch, Biotek, Bad Friedrichshall, Germany).

### Statistical analysis

Statistical analysis was performed using GraphPad Prism 9 software (La Jolla, CA, USA). Data were analyzed using One-way ANOVA with Tukey’s post-hoc correction. Statistically significant differences are indicated with asterisks and depicted as follows; *p* < 0.05 as *, *p* < 0.01 as **, *p* < 0.001 as ***, *p* < 0.0001 as ****.

**Table 1:** Primers used in this study.

Gene	Primer	Primer sequence 5’-3’	Amplicon Length (bp)	Accession No.
*SOD1*	F: R:	CATTCCATCATTGGCCGCAC GAGCGATCCCAATCACACCA	130	NM_001081826.3
*Nrf1*	F: R:	GGATTGTGCCTTCCATTCCTG TCCAATGTCACCACCTCCACA	193	XM_014739148.2
*SOD2*	F: R:	GGACAAACCTGAGCCCCAAT TTGGACACCAGCCGATACAG	125	NM_001082517.2
*CAT*	F: R:	ACCAAGGTTTGGCCTCACAA TTGGGTCAAAGGCCAACTGT	112	XM_014729341.2
*Nrf2*	F: R:	AAACCAGTGGGTCTGCCAA AATGAAGTCTGGGCTCTCGATG	188	XM_001496992.5
*GPX*	F: R:	TCGAGCCCAACTTCACACTC AAGTTCCAGGCGACATCGTT	178	NM_001166479.1
*Pink1*	F: R:	GCACAATGAGCCAGGAGCTA GGGGTATTCACGCGAAGGTA	298	XM_014737247.2
*PARKIN*	F: R:	CTGGAGGATTTAGTCCCGGAGC CCATGGCTGGAGTTGAACCTG	138	XM_005608126.3
*MIEF1*	F: R:	ATGCTGGGCATCGCTACAC CGGAGCCGTGACTTCTTCAA	284	XM_023631522.1
*PIGBOS*	F: R:	GTTGGGGTGGCTCAGATCAA ACCCCTCCTTTACCGCTACT	126	XM_014733689.2
*PPARGC1A*	F: R:	TCTACCTAGGATGCATGG GTGCAAGTAGAAACACTGC	74	XM_023638290.1
*PPARGC1B*	F: R:	CAACTATCTTGCCGACACCC ATGGGTTCAGTCTCGGGGTT	162	XM_023617445.1
*NDUFA9*	F: R:	TTGGTATTCAGGCCACACCC GCTGGCTTCACGTCTTCAAC	103	XM_001494601.4
*MRPL24*	F: R:	ATGATCCCTAGCGAAGCACC TGTAGAGACTCGTACCCGCT	123	XM_001500466.4
*PUSL1*	F: R:	TCAGCCACTTCCAGGACCTA AGCCACATCCAAGCTGTCTG	120	XM_023636049.1
*TFAM*	F: R:	ATGATGGCTTTGAGTCCAGG CTAGATGATGGCGGGAGACTT	154	XM_023643450.1
*OXA1L*	F: R:	GACCTAGAAACCGTGGGACG GGAAGATCACTTGGCTCCCC	105	XM_005603213.3
*UQCRC2*	F: R:	TGCTTCGTCTTGCATCCAGT AACTCCGGTGACGTGGTAAC	193	XM_001494381.5
*FIS*	F: R:	GGTGCGAAGCAAGTACAACG GTTGCCCACAGCCAGATAGA	118	XM_001504462.5
*GAPDH*	F: R:	GATGCCCCAATGTTTGTGA AAGCAGGGATGATGTTCTGG	250	NM_001163856.1

## Data Availability

The datasets generated during and/or analysed during the current study are available from the corresponding author on reasonable request.

## Abbreviations

PBMC: Peripheral blood mononuclear cells
ROS: Reactive oxygen species
RNS: Reactive nitrogen species
H_2_O_2_: Hydrogen peroxide
OS: Oxidative stress
